# A mechanistic model of methane emission from animal slurry with a focus on microbial groups

**DOI:** 10.1371/journal.pone.0252881

**Published:** 2021-06-10

**Authors:** Frederik R. Dalby, Sasha D. Hafner, Søren O. Petersen, Andrew Vanderzaag, Jemaneh Habtewold, Kari Dunfield, Martin H. Chantigny, Sven G. Sommer

**Affiliations:** 1 Department of Biotechnology and Chemical Engineering, Faculty of Technical Sciences, Aarhus University, Aarhus, Denmark; 2 Hafner Consulting LLC, Reston, Virginia, United States of America; 3 Department of Agroecology, Aarhus University, Tjele, Denmark; 4 Ottawa Research and Development Centre, Agriculture and Agri-Food Canada, Ottawa, Canada; 5 School of Environmental Science, University of Guelph, Guelph, Canada; 6 Quebec Research and Development Centre, Agriculture and Agri-Food Canada, Quebec, Canada; Tsinghua University, CHINA

## Abstract

Liquid manure (slurry) from livestock releases methane (CH_4_) that contributes significantly to global warming. Existing models for slurry CH_4_ production—used for mitigation and inventories—include effects of organic matter loading, temperature, and retention time but cannot predict important effects of management, or adequately capture essential temperature-driven dynamics. Here we present a new model that includes multiple methanogenic groups whose relative abundance shifts in response to changes in temperature or other environmental conditions. By default, the temperature responses of five groups correspond to those of four methanogenic species and one uncultured methanogen, although any number of groups could be defined. We argue that this simple mechanistic approach is able to describe both short- and long-term responses to temperature where other existing approaches fall short. The model is available in the open-source R package ABM (https://github.com/sashahafner/ABM) as a single flexible function that can include effects of slurry management (e.g., removal frequency and treatment methods) and changes in environmental conditions over time. Model simulations suggest that the reduction of CH_4_ emission by frequent emptying of slurry pits is due to washout of active methanogens. Application of the model to represent a full-scale slurry storage tank showed it can reproduce important trends, including a delayed response to temperature changes. However, the magnitude of predicted emission is uncertain, primarily as a result of sensitivity to the hydrolysis rate constant, due to a wide range in reported values. Results indicated that with additional work—particularly on the magnitude of hydrolysis rate—the model could be a tool for estimation of CH_4_ emissions for inventories.

## Introduction

Methane (CH_4_) emissions from livestock production make a significant contribution to global warming, and manure management on farms contributes about 6.5% of global anthropogenic CH_4_ emissions [1, 2]. Current emissions estimates in national inventories are based on guidelines from the IPCC [3], which offer a simple “Tier 1” approach with default emission factors for livestock categories and average annual temperature, and a more detailed “Tier 2” approach considering effects of organic matter (as volatile solids, VS) loading, retention time, and temperature, i.e., properties that vary with farming practices and location. Tier 2 estimates are currently based on a modification of the model presented by Mangino et al. [4], in which the fraction of VS converted to CH_4_ within each month is calculated from a van ’t Hoff-Arrhenius equation with an empirical estimate of activation energy and a reference point corresponding to 100% degradable VS conversion at 30 or 35°C. Although this provides a more site-specific estimate of CH_4_ emissions than fixed emission factors, the method has been found to poorly describe both temporal dynamics and total CH_4_ emissions in farm- and pilot-scale experiments [5–7]. Thus, a more accurate approach is needed to describe and quantify CH_4_ production in manure environments.

Models with a dynamic description of microbial decomposition of organic matter in anaerobic digesters already exist [8–11]. The ADM1 model was originally developed for anaerobic digestion almost two decades ago [10], and it remains a useful and popular tool for research and possibly even plant management [12, 13]. Despite its complexity (at least 26 differential equations), ADM1 and similar models were not developed to predict responses to temperature change known to affect CH_4_ production in stored slurry. The distinction between short- and long-term responses to environmental changes is also not included in these or most other models, which therefore cannot be used to assess slurry management practices such as cooling as a means to reduce CH_4_ emissions, or even for accurate estimation of seasonal variations. For example, an empirical model that accounted for daily temperature and VS degradation still failed to capture the observed dynamics of CH_4_ emissions, and it was concluded that the description of methanogenic activity under variable slurry storage conditions was inadequate [5].

**Fig 1:**
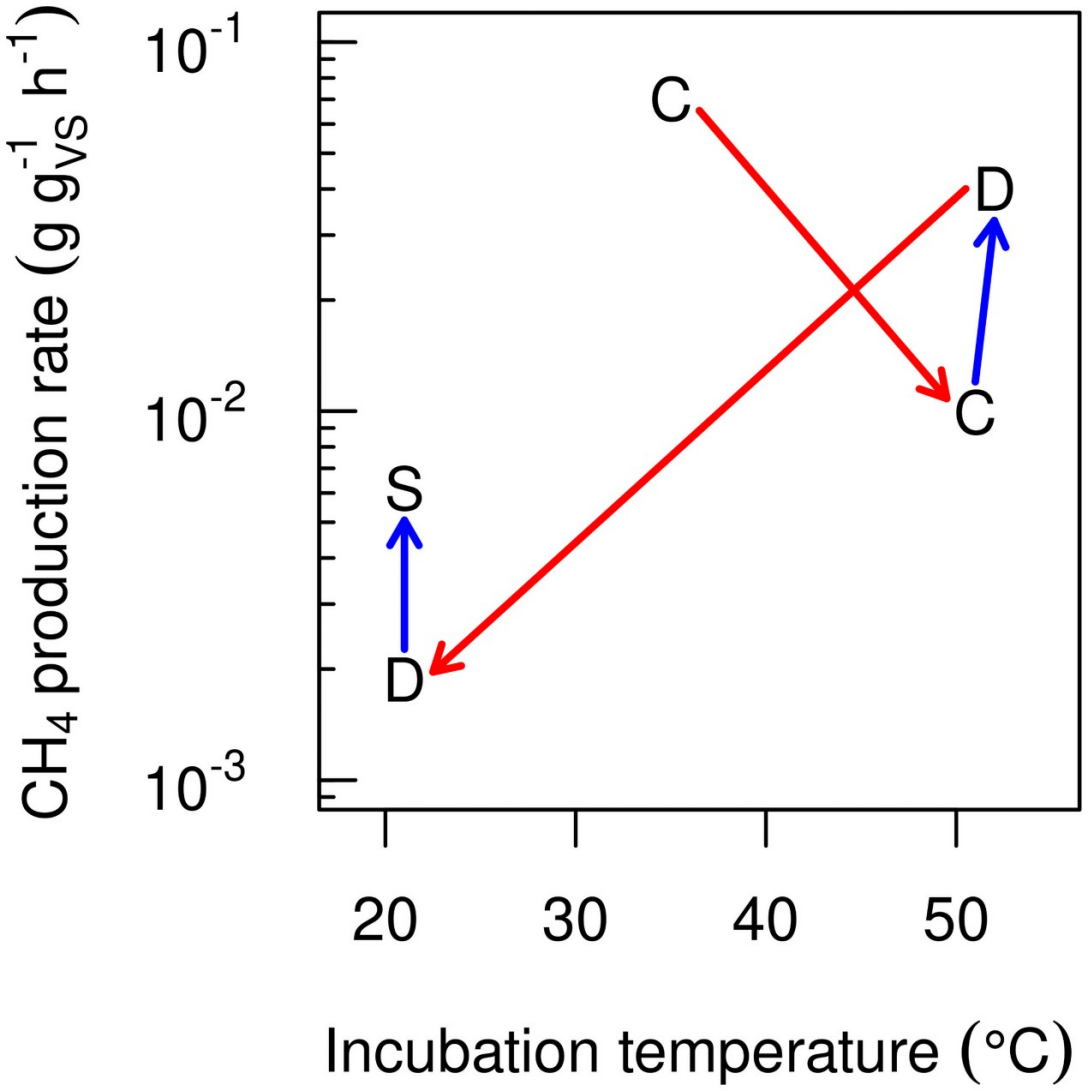
Example of short- and long-term responses of methane production to temperature change. Differences between short- and long-term response to temperature change measured by Elsgaard et al. [22]. Labels identify source: C = cattle manure (from barn), D = fresh digestate (directly from anaerobic digester), S = stored (> 1 month) digestate. Red arrows show short-term effects (differences between samples from the same source when incubated for 17 hours), and blue arrows apparent long-term effects (differences for samples stored for short and long time (weeks or months) at the same temperature)).

In storage experiments with both fresh and aged slurry, a period of days to months with low CH_4_ emission rates has often been observed [5, 14–18]. Such a lag phase may reflect the time required for substrates of methanogenesis to reach a threshold concentration supporting growth, or the time required for development of an adapted methanogenic community. Some studies have highlighted the importance of residual aged manure in a storage acting as an inoculum, which suggests that community development is central for the temporal dynamics of CH_4_ emissions [19–21]. Thus, short-term changes due to temperature variation may reflect the activity of an existing methanogenic community, while long-term changes include the effects of successional changes of the community. Recent measurements of CH_4_ production rates in manure and digestate at temperatures between 5 and 52°C [22] highlight the difference between short- and long-term responses. These measurements show that the short-term (hours to days) response to a change in temperature is generally a shift away from the optimum of the active methanogenic community, but over time the activity at the new temperature will increase (Fig 1). Although some of the long-term differences in CH_4_ production were undoubtedly due to changes in substrate availability, considering them would tend to magnify the differences between short- and long-term responses. The general trend shown in Fig 1 is typical in studies of temperature change during anaerobic digestion [23–26].

The distinction between short- and long-term temperature responses was recently quantitatively addressed through development of an anaerobic digester model that included gradual changes in the temperature optimum of kinetic parameters for a single population of methanogens [27]. However, an empirical approach was used that does not explicitly represent the underlying mechanism. Gene sequencing has revealed that temperature changes shift the relative abundance of taxonomic groups of methanogens, indicating that changes in CH_4_ production rates are due to selective growth of adapted methanogenic populations, rather than adaptation of an already established consortium [24, 28, 29]. Presumably the response to environmental stresses other than temperature also varies among methanogenic populations [30–32], and accordingly models need to include multiple groups of methanogens with different responses to temperature, and perhaps other stressors, in order to accurately predict both lag phases and the difference between short- and long-term response to changes in temperature and the chemical environment. This discussion also highlights an important challenge for prediction of CH_4_ emission: measurements of short-term temperature responses in the laboratory, however careful, may not reflect long-term seasonal or geographic responses that are important for total CH_4_ emissions.

Besides methanogens, there is evidence that sulfate reducing bacteria can affect CH_4_ emission by competing for substrate (acetate or hydrogen) [33, 34], or by the production of inhibitory hydrogen sulfide (H_2_S) [35, 36]. This is particularly important for acidification of liquid manure with sulfuric acid, where prolonged suppression of CH_4_ emission has been observed, even when pH returned to or remained at near-neutral [15, 37]. In the absence of suitable electron acceptors, processes other than methanogenesis are not expected to play a major role beyond fermentation in this anaerobic environment [38]. Although sulfate may be used to oxidize ammonia [39], it is unlikely that this autotrophic process is important in organic-rich slurry. At the slurry-air interface, there is a potential for production of nitrous oxide (N_2_O) through nitrification and denitrification [40], as well as for bacterial methane oxidation [41, 42], but in both cases this depends on the development of a partly dry surface crust. While the model described below includes oxidation of organic matter in the surface layer, a crust represents a different environment that is outside the scope of the presented model. Still, these processes are part of a complete assessment of greenhouse gas emission from livestock operations.

Existing mechanistic models of organic matter degradation in anaerobic digesters describe biochemical pathways in detail, but are difficult to apply to highly variable manure environments due to lack of data. For example reported hydrolysis rate constants for slurry vary between 0.004 and 0.13 d^-1^ [43–45], possibly due to effects of feeding practice, manure management, or manure age [43, 46]. The main substrates for methanogenesis in slurry are volatile fatty acids (VFAs) and hydrogen [47, 48], but hydrogen is mainly derived from VFA oxidation [49, 50], and its regulatory role may be exaggerated [51]. Consequently, some models and simplified versions of detailed models merge hydrogen and VFA consumption kinetics to more simply represent organic matter degradation pathways [8, 11].

The considerations presented above strongly support the need for a new approach to predict CH_4_ emission from stored manure that describes both short- and long-term responses to management and storage conditions—including temperature and substrate availability—more accurately than current models. We propose that these responses can be accurately described using a simple dynamic model that includes multiple methanogenic populations with different temperature responses. Both inhibition and competition can easily be incorporated into this framework. The objective of the study was to develop and implement this approach as a new mechanistic model that can be used to better understand and predict of CH_4_ emission from slurry storage environments, including pits or channels inside barns and outside storage facilities.

## Methods

The new model presented in this paper predicts slurry organic matter transformation to CH_4_ using chemical oxygen demand (COD) as the base unit, though with conversion to a volatile solids (VS) basis for convenience. Carbon dioxide (CO_2_) production is also predicted, for a complete mass balance (or carbon balance) and also because it is a greenhouse gas. The essential model components are given below; further justification for these choices are presented in a related review [52].

1. Organic matter (volatile solids) includes three degradable components (particulate material, volatile fatty acids (VFAs), and microbial biomass) and a single non-degradable component, which is assumed to be completely conserved.
2. A single first-order expression for disintegration/hydrolysis and fermentation is used, reflecting the assumption that hydrolysis (but typically not fermentation) may limit the rate of CH_4_ production in slurry [43, 44, 53].
3. A Monod expression with VFAs as substrate is used for calculating microbial activity. This approach was chosen because Monod parameters are most frequently reported in the literature [11].
4. Multiple methanogen groups and one group of sulfate reducers, all with individual responses to temperature, are defined in order to capture microbial dynamics and short- and long-term changes in activity in response to temperature changes.
5. Management options that control slurry production- and removal rates are integrated to account for microbial adaptation in outside storages or in-house pits or channels.
6. Microbial inhibition factors are included for pH, free ammonia (NH_3_ (aq)) and ammonium (NH_4_^+^), and hydrogen sulfide (H_2_S).

### Model processes and algorithms

**Fig 2:**
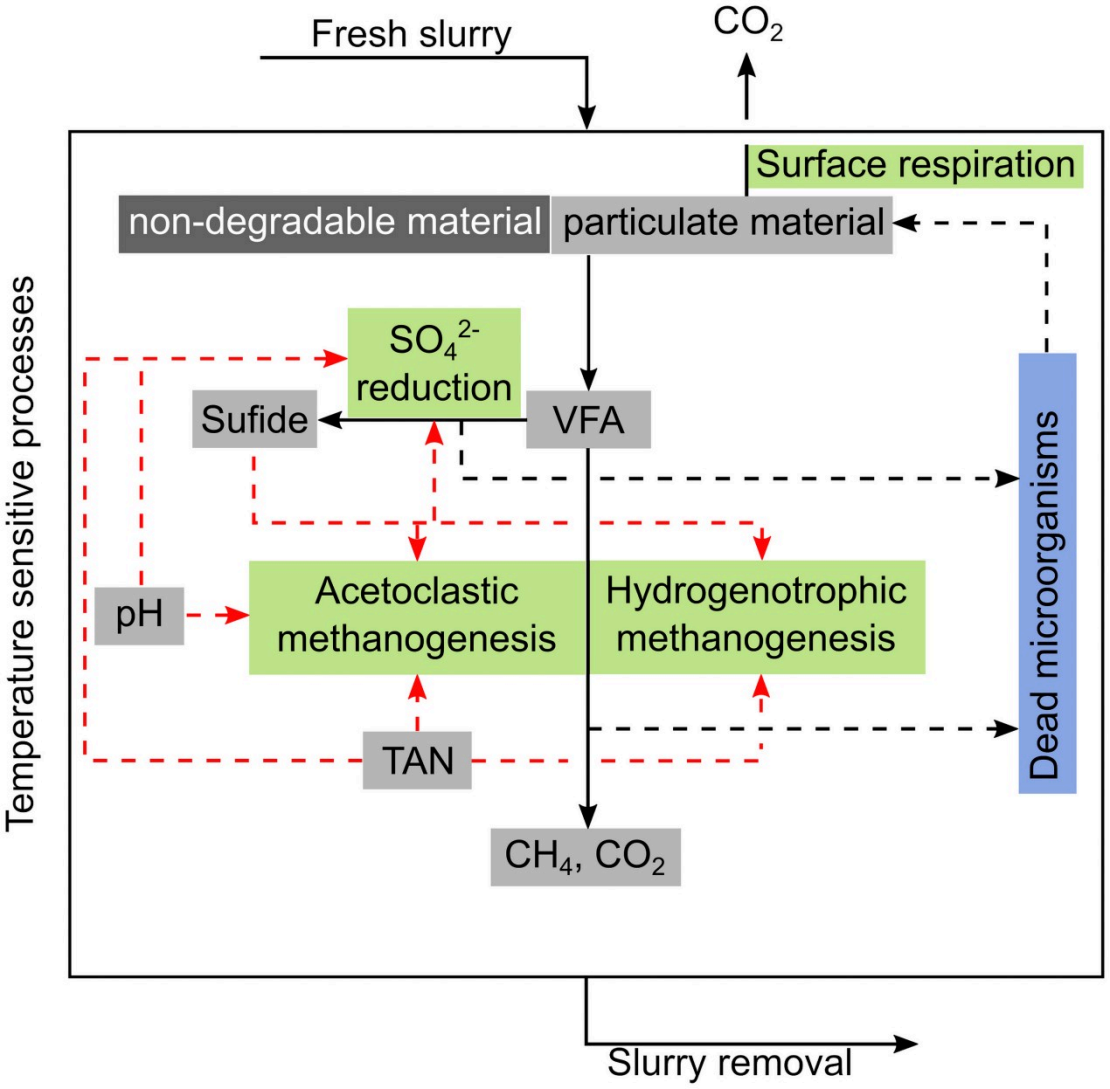
Flow diagram of model concept. Black lines indicate flows of organic matter, black dashed lines indicate flows of decayed microbial biomass. Red dashed lines indicate factors that inhibit microbial conversion processes (green boxes). Temperature affects all conversion processes, as well as chemical speciation.

A flow diagram of the key processes included in the model are shown in Fig 2, and Table 1 summarizes processes and rates expressions.

**Table 1:** Petersen matrix of model state variables (following [61], based on [62]). See S3 Appendix for parameter descriptions and default values.

	Component → *j*	1	2	3	4	5	6	7	8	Rate expression
***i***	**Process ↓** ^c^	**S**_**p**_	**VFAs**	**SO**_**4**_^**2-**^	**sulfide**	**CH**_**4**_	**CO**_**2**_	**X**_**i**_	**X**_**sr**_	
**1**	**Hydrolysis & fermentation**	-1	1							*α* · *S*_*p*_
**2**	**Methanogenesis of X**_**i**_ ^a^		-1			$P_{{\text{CH}}_{4}}$	$P_{{\text{CO}}_{2},\;anaer}$	*Y*_*i*_		$\frac{q_{max}\;∙C_{VFA}}{K_{S}+C_{VFA}}∙X_{i}∙I_{i}$
**3**	**Sulfate reduction of X**_**sr**_ ^a^		-1	*f*_COD–S,sulfur_^b^	*f*_COD–S,sulfur_^b^		$P_{{\text{CO}}_{2},\;sr}$		*Y*_*sr*_	$\frac{q_{max}∙C_{VFA}}{K_{S}+C_{VFA}}∙\frac{C_{{SO}_{4}}}{K_{S,{SO}_{4}}+C_{{SO}_{4}}}\;X_{sr}∙I_{sr}$
**4**	**Surface respiration**	-1					$P_{{\text{CO}}_{2},\;aer}$			$k_{L,{\text{O}}_{2}}∙area∙(0.208k_{H,{\text{O}}_{2}}-0)$
**5**	**Hydrogen sulfide emission**				-1					${kL}_{H_{2}S}∙area∙(C_{H_{2}S}-0)$
**6**	**Decay of X**_**i**_ ^a^	1						-1		*k*_*d*,*i*_ · *X*_*i*_
**7**	**Decay of X**_**sr**_ ^a^	1							-1	*k*_*d*,*sr*_ · *X*_*sr*_
**8**	**Slurry addition**	*F*_*in*_	*F*_*in*_	*F*_*in*_	*F*_*in*_	*F*_*in*_		*F*_*in*_	*F*_*in*_	*C*_*si*,*in*_ *or C*_*Xi*,*in*_
		Degradable organic matter (g_COD-S_)	Volatile fatty acids (g_COD-S_)	Sulfate (${\text{g}}_{{\text{SO}}_{4}-\text{sulfur}}$)	Sulfide (${\text{g}}_{{\text{H}}_{2}\text{S}-\text{sulfur}}$)	Methane (${\text{g}}_{{\text{CH}}_{4}}$)	Carbon dioxide (${\text{g}}_{{\text{CO}}_{2}}$)	Methanogen populations (g_COD-B_)	Sulfate reducer populations (g_COD-B_)	rate units *i* = 1–4 (g_COD-S_ d^-1^) *i* = 5 (${\text{g}}_{{\text{H}}_{2}\text{S}-\text{sulfur}\;{\text{d}}^{-1}}$) *i* = 6,7 (g_COD-B_ d^-1^) *i* = 8 (g_COD-S_, g_COD-B_, ${\text{g}}_{{\text{H}}_{2}\text{S}-\text{S}},\;{\text{g}}_{{\text{SO}}_{4}-\text{S}}$, g_TAN_) d^-1^

^**a**^ Can represent any number of microbial groups.

^**b**^ Unit conversion factor.

^**c**^ Slurry and components removal are not included in this table since these do not occur by rate. Instead see Eqs 8–11.

Disintegration and hydrolysis of particulate material are considered rate limiting for subsequent degradation. Hence, combined disintegration, hydrolysis, and fermentation of degradable particulate material to VFAs is calculated with first order kinetics [43–45] (Eq 1).


$$\frac{dS_{p}}{dt}=-\;\alpha ∙S_{p}-R+\sum_{i=1}^{k}{(k}_{d,i}∙X_{i})+F_{in}∙\;C_{S_{p},in}$$ (1)

In Eq 1, *α* is the first-order rate constant (d^-1^), *S*_*p*_ is degradable particulate material in units of substrate COD (g_COD-S_), and *t* is time (d). Surface respiration rate, *R* (g_COD-S_ m^-2^ d^-1^), reduces *S*_*p*_ but model predictions suggest it is only significant when the slurry depth is a few mm. Dead microbial biomass from any defined microbial group (*i*) is reintroduced as *S*_*p*_, where *k*_*d*,*i*_ (d^-1^) is the decay rate constant of the microbial groups. *X*_*i*_ represents the active biomass (g_COD-B_) of microbial group *i*, with a total of *k* groups, all methanogens except for 1 sulfate reducer. *F*_*in*_ is the slurry production rate (kg_slurry_ d^-1^), and *C*_*Sp*,*in*_ is the *S*_*p*_ concentration of the introduced slurry (g_COD-S_ kg_slurry_^-1^). Hydrolysis of *S*_*p*_ yields VFAs (g_COD-S_), which in turn is consumed by microbial groups (Eq 2).


$$\frac{dVFA}{dt}=\alpha ∙S_{p}-\sum_{i=1}^{k}r_{i}+F_{in}∙\;C_{VFA,in}$$ (2)

Here, *r*_*i*_ is the VFA utilization rate (g_COD-S_ d^-1^), which follows Monod kinetics, and *C*_*VFA*,*in*_ is the VFA concentration (g_COD-S_ kg_slurry_^-1^) in the introduced slurry. The VFA utilization rate of each methanogen group *r*_*i*_ is linked to the active biomass (Eq 3) [38].


$$r_{i}=-\frac{q_{max,i}\;∙C_{VFA}}{K_{S,i}+C_{VFA}}∙X_{i}∙\prod_{j=1}^{4}I_{i,j}$$ (3)

Here *q*_*max*_ is the temperature-dependent maximum specific substrate utilization rate (g_COD-S_ g_COD-B_^-1^ d^-1^), *K*_*S*_ is the half-saturation constant (g_COD-S_ kg_slurry_^-1^), and *C*_*VFA*_ is the concentration of VFAs in the slurry (g_COD-S_ kg_Sslurry_^-1^), and *X*_*i*_ represents the active methanogen biomass of group *i*. *I*_*i*,*j*_ is a dimensionless inhibition term with a value between 0 and 1 that represents the sensitivity of group *i* to inhibitor *j* with a total of 4 potential inhibitors (Table 2). The substrate utilization rate for sulfate reducers, *r*_*sr*_, follows a double Monod expression (Eq 4) [36].

**Table 2:** Equations used for inhibition factors.

Inhibition factor	Expression	Description	Reference
$I_{{NH}_{3}}$	$\{\begin{array}{cc} 1, & C_{{NH}_{3}}\leq {KI}_{NH3,min}\\ e^{-2.77259∙{\left(\frac{C_{NH3}-{KI}_{NH3,min}}{{KI}_{NH3,max}-{KI}_{NH3,min}}\right)}^{2}}, & C_{{NH}_{3}}>{KI}_{NH3,min} \end{array}$	Inhibition factor for ammonia, where ${KI}_{{NH}_{3},min}$ and ${KI}_{{NH}_{3},max}$ are the NH_3_ concentration at which inhibition starts and reach maximum, respectively	[63]
$I_{{NH}_{4}}$	$\{\begin{array}{cc} 1, & C_{{NH}_{4}}\leq {KI}_{{NH}_{4},min}\\ e^{-2.77259∙{\left(\frac{C_{NH4}-{KI}_{NH4,min}}{{KI}_{NH4,max}-{KI}_{NH4,min}}\right)}^{2}}, & C_{{NH}_{4}}>{KI}_{{NH}_{4},min} \end{array}$	Inhibition factor for ammonium, where ${KI}_{{NH}_{4},min}$ and ${KI}_{{NH}_{4},max}$ are the NH_4_^+^ concentrations at which inhibition starts and reach 100% inhibition, respectively	[63]
*I*_*pH*_	$\frac{1+2∙10^{0.5({pH}_{L}-{pH}_{U})}}{1+10^{\left(pH-{pH}_{U}\right)}+10^{\left({pH}_{L}-pH\right)}}$	Inhibition factor for pH, with upper (*pH*_*U*_) and lower (*pH*_*L*_) pH limits at which 50% inhibition occurs.	[8]
$I_{H_{2}S}$	$\{\begin{array}{cc} 1-\frac{C_{H_{2}S}}{{KI}_{H_{2}S}}, & C_{H2S}\leq \;{KI}_{H_{2}S}\\ 0, & C_{H2S}>\;{KI}_{H_{2}S} \end{array}$	Inhibition factor for H_2_S, where ${KI}_{H_{2}S}$ is the H_2_S concentration at which 100% inhibition occurs.	[36]
*I*	$I_{{NH}_{3}}∙I_{{NH}_{4}}∙I_{pH}∙I_{H_{2}S}$	Joint inhibition factor	[11]


$$r_{sr}=-\frac{q_{max\;}\;∙C_{VFA}}{K_{S}+C_{VFA}}∙\frac{C_{SO_{4}}}{K_{S,SO_{4}}+C_{SO_{4}}}\;X_{sr}∙\prod_{j=1}^{4}I_{sr,j}$$ (4)

where $C_{SO_{4}}$ and $K_{S,SO_{4}}$ are the concentration of SO_4_^2-^ (${\text{g}}_{\text{S}{\text{O}}_{4}-\text{S}}\;{\text{kg}}_{\text{slurry}}^{-1}$) and the half-maximum SO_4_^2-^ saturation constant (${\text{g}}_{\text{S}{\text{O}}_{4}-\text{S}}\;{\text{kg}}_{\text{slurry}}^{-1}$), respectively. (Note that charges on chemical species are omitted in subscripts for clarity in text and tables, e.g., SO_4_ for sulfate.) *X*_*sr*_ represents the biomass of active sulfate reducing bacteria. Sulfate is reduced to sulfide in a 1:1 molar ratio, and loss of H_2_S to the air is proportional to the slurry surface area (*A*). Microbial growth of any group is linked to *r*_*i*_ through the biomass/substrate yield coefficient *Y*_*i*_ (g_COD-B_ g_COD-S_^-1^), and the biomass decay follows first order kinetics (Eq 5).

$$\frac{dX_{i}}{dt}=Y_{i}∙r_{i}-k_{d,i}∙X_{i}+F_{in}∙C_{Xi,in}$$ (5)

*C*_*Xi*,*in*_ is the concentration of active microbial biomass (${\text{g}}_{\text{COD}-\text{B}}\;{\text{kg}}_{\text{slurry}}^{-1}$) in the fresh slurry. Methane production is linked to substrate utilization *r* for methanogens using a CH_4_ productivity coefficient, $P_{{CH}_{4}}\;({\text{g}}_{\text{C}{\text{H}}_{4}}\;{\text{g}}_{\text{COD}-\text{S}}^{-1}$), so CH_4_ production rate (${\text{g}}_{\text{C}{\text{H}}_{4}}\;{\text{d}}^{-1}$) is given by Eq 6.


$$\frac{dCH_{4}}{dt}=P_{{CH}_{4}}∙\sum_{i=1}^{k-1}r_{i}$$ (6)

The CO_2_ production is linked to microbial activity through productivity coefficients (g_CO2_ g_COD-S_^-1^) in Eq 7.


$$\frac{dCO_{2}}{dt}=P_{{\text{CO}}_{2},anaerobic}∙\sum_{i=1}^{k-1}r_{i}+P_{{CO}_{2},sr}∙r_{sr}+P_{{CO}_{2},\;aerobic}∙R$$ (7)

Because $P_{{\text{CO}}_{2},anaerobic}$ includes CO_2_ from both fermentation and methanogenesis, it is only accurate when the system is in steady state, or as a cumulative response, and less so during VFA accumulation.

Slurry is added at a constant rate until the storage reaches maximum capacity (Eq 8), at which time slurry is removed, resulting in a new level (Eq 9).


$$\frac{dM_{m}}{dt}=F_{in}$$ (8)

$$M_{m}^{\prime }=M_{m}∙f_{resid}$$ (9)

Slurry removal is instantaneous and occurs exactly when slurry mass *M*_*m*_ equals *M*_*m*,*max*_. $M_{m}^{\prime }$ is the slurry mass after removal, and *f*_*resid*_ is the fraction of slurry retained in the channel after removal. The total mass of *S*_*p*_, VFAs, SO_4_^2-^, TAN, and H_2_S are similarly reduced.

Methanogen enrichment in residual slurry is probable in the light of documentation that methanogenic biofilms form on various materials under a range of environmental conditions [54–56]. To account for this, a microbial enrichment factor *a*_*enrich*_, representing the increase in the odds of retention for a single microorganism relative to the odds for conservative components, was used (Eqs 10 and 11). An *a*_*enrich*_ value of zero implies no enrichment.


$$f_{resid,Xi}=\frac{e^{ln(\frac{fresid}{{1-f}_{resid}})+a_{enrich}}}{1+e^{ln(\frac{fresid}{{1-f}_{resid}})+a_{enrich}}}$$ (10)

$$X_{i}^{\prime }=X_{i}∙f_{resid,Xi}$$ (11)

Here, *f*_*resid*,*Xi*_ is the fraction of a single population retained after slurry removal and $X_{i}^{\prime }$ is the microbial biomass (g_COD-B_) of this group after slurry removal.

Temperature sensitivity of *q*_*max*_ and *α* is based on the Cardinal Temperature Model (CTM1) [57, 58] shown for *q*_*max*_ in Eq 12.

$${q_{max}=q}_{max,opt}\frac{(T-T_{max})∙{\left(T-T_{min}\right)}^{2}}{\left(T_{opt}-T_{min})∙[\left({T_{opt}-T}_{min})∙(T-T_{opt})-(T_{opt}-T_{max})∙(T_{opt}+T_{min}-2∙T\right)\right]}$$ (12)

*q*_*max*_ is calculated for each microbial population (group) by selecting the following temperature constraints on substrate utilization; minimum temperature of substrate utilization, *T*_*min*_ (°C), maximum temperature of substrate utilization, *T*_*max*_ (°C), the optimum temperature of substrate utilization, *T*_*opt*_ (°C), the temperature of the slurry material, *T* (°C), and the maximum substrate utilization rate at *T*_*opt*_, *q*_*max*,*opt*_ (g_COD-S_ g_COD-B_^-1^ d^-1^). Traditionally, temperature effects on metabolic processes are described with a double Arrhenius expression [59]. The CTM1 model predicts a similar response and uses intuitive temperature inputs (*T*_*max*_, *T*_*min*_, *T*_*opt*_ and *T)*, which allows for easy tuning of microbial growth characteristics. *K*_*S*_ decreases with temperature according to an exponential function [60] (Eq 13).


$$K_{S}={K_{S,coef}∙k}_{1}\text{exp}(-k_{2}∙T)$$ (13)

The processes described above all affect state variables of the model, which are summarized in Table 1. Growth inhibition factors are presented in S2 Appendix. Temperature-dependent equations describing chemical speciation and air-slurry transfer of H_2_S and O_2_ are provided in S2 Appendix.

### Methanogenic groups

**Fig 3:**
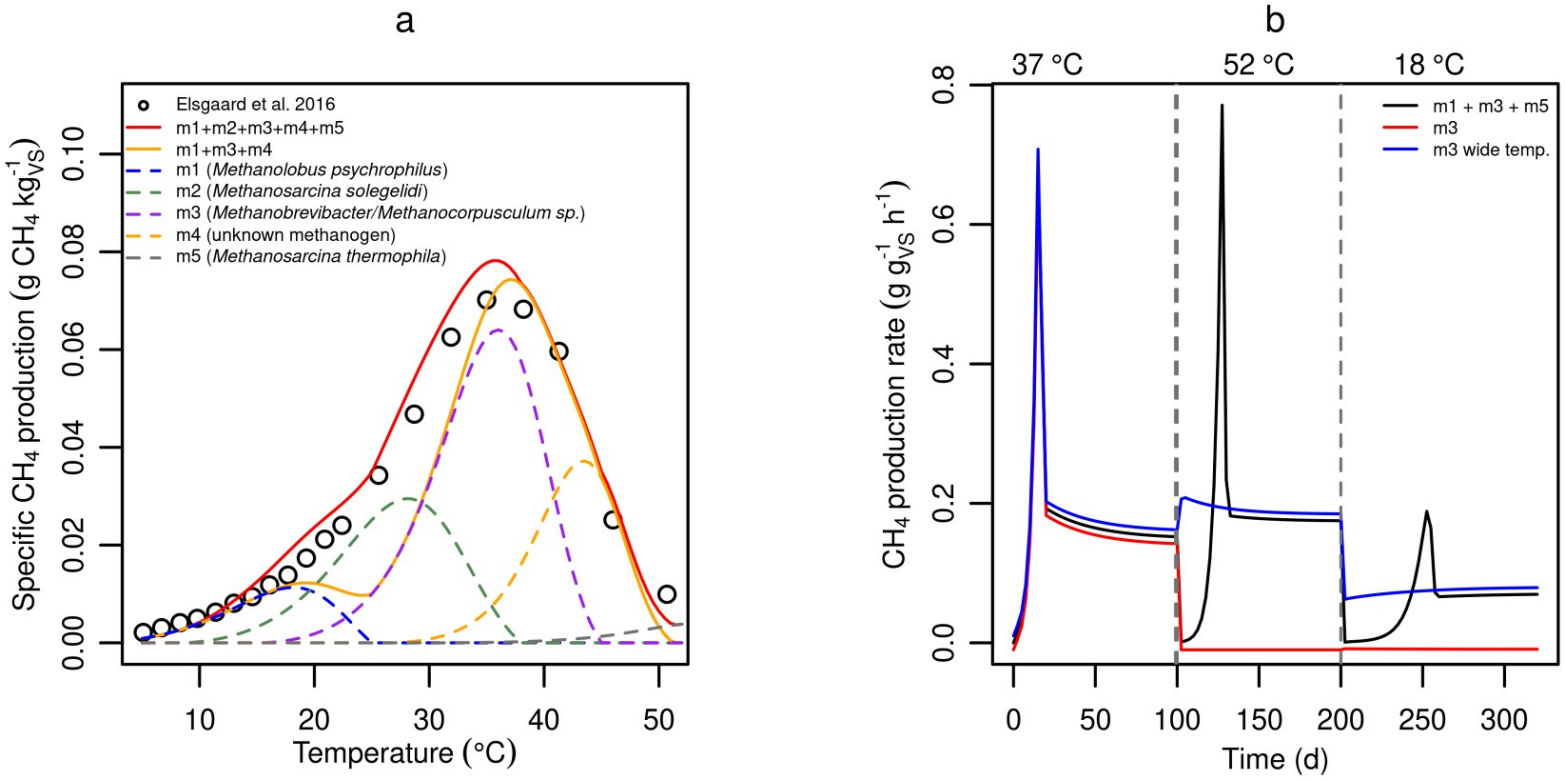
Number of methanogen groups. **(a)** Predicted CH_4_ production of single (dashed colored lines) and multiple (solid lines) methanogen groups as compared to observed CH_4_ production in cattle slurry by Elsgaard et al 2016 (circles). **(b)** Predicted short- and long-term responses to temperature change for different sets of methanogen groups. Note: Lines are shifted up or down by 0.01 for clarity.

The proposed model can accept any number of methanogenic groups, but for simplicity it should include only those necessary for reproducing important short- and long-term responses. Although this set may vary with application [31, 64], here we present a generic set based on the short-term temperature response for cattle manure presented by Elsgaard et al. [22], along with pure culture results from Jabłoński et al. [65]. Elsgaard et al. [22] studied CH_4_ production rates from slurry and digestate during incubation for up to two days at temperatures between 5 and 52°C and found methanogenic activity over the whole temperature range. Considering that the average temperature growth interval of methanogenic species in the database compiled by Jabłoński et al. [65] is only 25°C (*n* = 104), observation of CH_4_ production over 47°C by Elsgaard et al. [22] suggest that multiple methanogen groups were present and active in the manure materials. Fig 3a shows the increased modelling accuracy achieved with multiple methanogen groups using temperature response profiles selected based on methanogenic community analysis studies of cattle slurry [48, 64, 66–68] and the methanogenic database [65]. At least five groups (red full line, with default parameter values for m1, m2, m3, m4, and m5) are required to match measured CH_4_ production over the whole temperature range. Thus, five groups that approximately represent individual methanogen species (with the exception of m4) were selected as the default set.

Although a single methanogen group could probably capture observed short-term responses, it is necessary to include multiple groups of methanogens to account for general differences between short- and long-term measurements (e.g., Fig 1). In Fig 3b the differences between a single and multiple methanogen groups in terms of short-and long-term effects are clearly demonstrated. The expected difference between short- and long-term responses is achieved only with multiple groups (black, with default parameter values for m1, m3, and m5). A single methanogen group, even with an extremely wide temperature growth interval, shows only minor differences in short vs long-term effects on CH_4_ production after a temperature change. On the other hand, with multiple methanogenic groups there is a large difference, where the short-term effect is a drop of CH_4_ emission followed by an increase and stabilization period in the long-term. This response is consistent with observations in multiple experiments [5, 14–17, 69] and confirms the importance of including multiple methanogen groups for accurate modelling of CH_4_ emission of slurry storage systems.

### Model parameters

**Fig 4:**
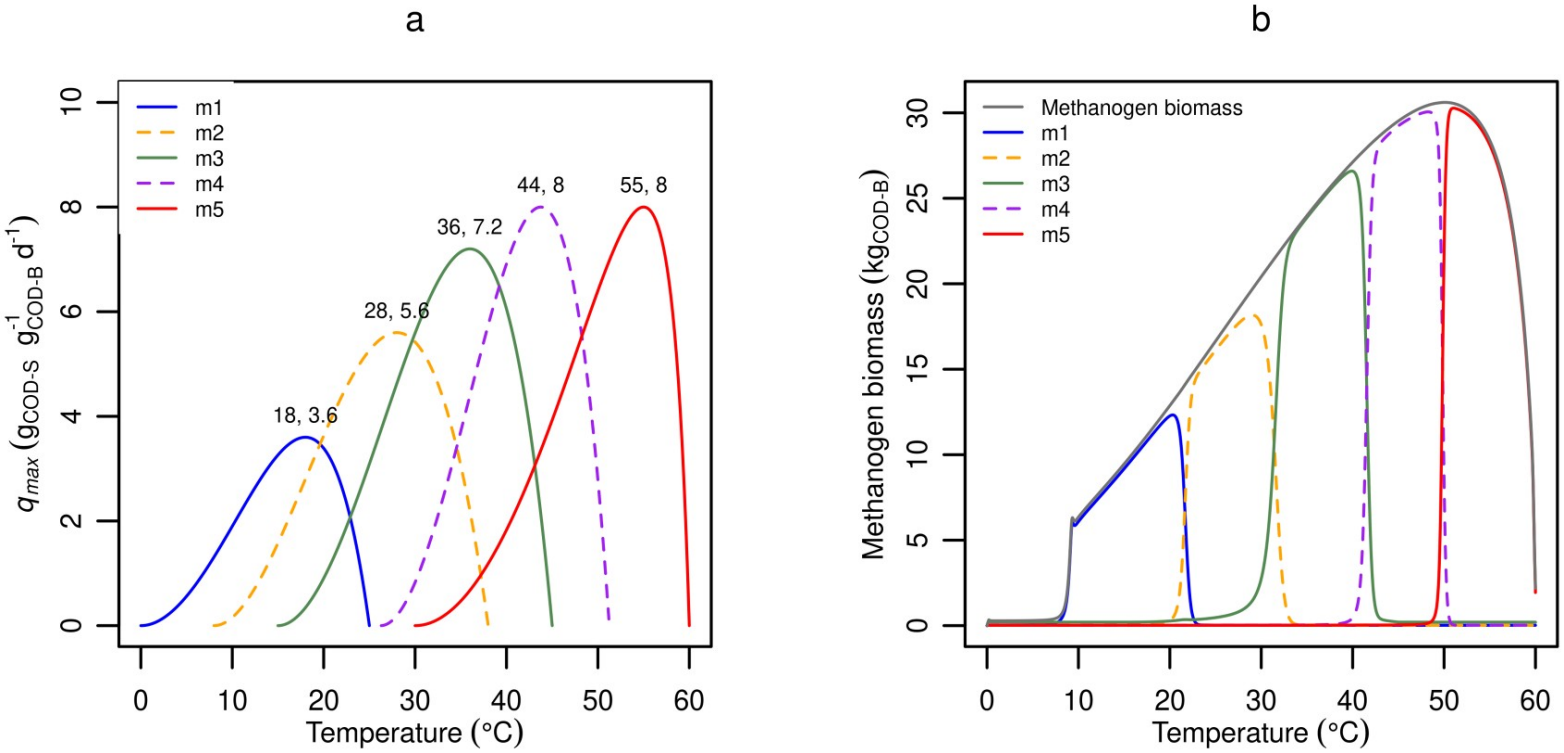
Default temperature responses. **(a)** Temperature dependence of maximum substrate utilization rates (*q*_*max*_) of default methanogen groups in the model (m1 to m5). **(b)** Steady-state microbial biomass as a function of temperature for default parameter values. The residual fraction of slurry (*f*_*resid*_) was set to 0.95 in this simulation.

Based on Fig 3, five methanogen groups (m1—m5) were chosen as default. Temperature optima of these groups are based on individual species: *Methanolobus psychrophilus* (m1), *Metahnosarcina solegelid* (m2), *Metahnobrevibacter/Methanocorpusculum* sp. (m3), and *Methanosarcina thermophilia* (m5). The final group (m4) is not based on a known species, but instead has an optimum temperature equidistant between that of m3 and m5. The *q*_*max*,*opt*_ of each group was calculated from its optimum growth temperature shown in Fig 4a, by assuming a linear increase from 0 g_COD-S_ g_COD-B_^-1^ d^-1^ at 0°C to 8 g_COD-S_ g_COD-B_^-1^ d^-1^ at 40°C. In comparison, by default ADM1 uses 8 g_COD-S_ g_COD-B_^-1^ d^-1^ at 35°C [11]. The temperature dependent *q*_*max*_ for individual populations (Fig 3a) is calculated using *q*_*max*,*opt*_ according to Eq 12. For all methanogen groups, biomass yield *Y*_*i*_ was fixed at 0.05 g_COD-B_ g_COD-S_^-1^, equal to the recommended value for acetoclastic methanogen in ADM1 [10] and the mean value from the literature review presented by Weinrich [70, 71] (*n* = 37 measurements). The *q*_*max*,*opt*_ and *Y*_*i*_ of the optional sulfate reducer group, sr1, were calculated based on the relative *q*_*max*,*opt*_ and *Y*_*i*_ difference between typical acetoclastic methanogens and acetoclastic sulfate reducers [38]. Hydrogenotrophic methanogens such as *Methanobrevibacter* and *Methanocorpusculum* (m3) are highly abundant in fresh manure and in the animal intestinal tract [48, 65, 72, 73], and hence the m3 active biomass concentration in the produced slurry is by default an order of magnitude greater than for any other methanogen group (0.01 vs 0.001 g_COD-B_ kg_Slurry_^-1^). The *K*_*s*_ was calculated using Eq 13, where *K*_*S*,*coef*_ by default is 1, but can be modified for individual microbial groups to reflect differences in substrate affinity for e.g. acetoclastic methanogens, hydrogenotrophic methanogens [74–76] and sulfate reducers [33, 34]. For the same reasons, inhibition constants can be specified for individual microbial groups [36, 77]. The temperature dependent disintegration/hydrolysis/fermentation rate constant, *α*, was calculated from Eq 12 with default *α*_*opt*_ = 0.02 d^-1^ at *T*_*opt*_ = = 50, *T*_*min*_ = = 0, and *T*_*max*_ = = 60°C. Productivity coefficients for CH_4_ and CO_2_ were calculated based on microbial stoichiometry (see S1 Appendix) [38, 78]. The default value of a mass transfer coefficient for O_2_ was based on respiration rates measured by Markfoged [79], and for H_2_S, was estimated by assuming depletion occurred within a 1 cm film at the surface. The complete list of default parameter values and input variables are found in S3 Appendix.

Fig 4b shows the model-predicted steady state abundance of the default microbial groups as a function of temperature. The steady state active methanogen biomass smoothly increases with temperature above about 10°C, despite shifting dominance among methanogen groups. This correlation is an indirect consequence of higher *q*_*max*_ and *α* with increasing temperature. However, low *q*_*max*_ below 10°C translates into very limited growth of m1, which is consistent with small CH_4_ emissions reported at low temperatures [6, 22]. Above 10°C the total methanogen biomass curve resembles the shape of the hydrolysis rate curve (Eq 12), because hydrolysis (i.e., substrate availability and not maximum methanogen growth rates) is rate limiting in this simulation, due to the emptying interval and residual fraction (see “Model behavior” section).

### Model behavior and application

The model was implemented in the R language [80] as a function, and is available as an add-on package from GitHub at https://github.com/sashahafner/ABM. A vignette included with the package provides an introduction to the use of the model. The abm() function in this package (version 1.18.0) was used to generate results shown in this work.

Effects of slurry retention time, temperature change, and pH were explored in order to show the behavior of the model. Simulations were generally run using the default parameter settings, with a slurry production rate of 1000 kg d^-1^ and a slurry storage capacity of 33333 kg, equivalent to conditions of a slurry channel or pit receiving fresh excreta, and being emptied (except for a 10% residual fraction) every 30 days. The default temperature is 20°C and pH is 7. Below, deviations from default parameters are explicitly stated and listed in S3 Appendix. In addition, we present a sensitivity analysis, and a comparison of model simulations to measurements from a farm scale experiment. To show the capability of the model to describe CH_4_ emissions from real livestock production facilities, we compared model simulations to data from Kariyapperuma et al. [6], who measured CH_4_ emission from an outside slurry storage tank with a maximum capacity of about 2700 m^3^. The slurry storage tank periodically received manure from a pre-storage underneath a barn with approximately 200 cows. For additional details on the manure management see [6]. The dataset lacked information about the initial and subsequently introduced slurry composition, and instead we made qualified guesses and ran multiple simulation scenarios to assess model performance. The dataset included time series of organic matter concentration in units of VS, and hence conversion to degradable particulate material (COD units) was needed to make it compatible with the model input unit. Since COD measurement is error-prone for particulate materials [81], use of a conversion factor to estimate COD from VS is preferable to direct measurement. The degradable fraction of VS (VS_d_) in cattle slurry was set to 0.42, based on the average of two studies [82, 83]. The COD-to-VS ratio was estimated at 1.42 g_COD-S_/g_VS_ for cattle slurry based on average slurry composition [82] and calculated oxygen demand [38] (S1 Appendix). We assumed that half the VS_d_ had been degraded at the start of the measurements, based on recent estimates suggesting that 28% of VS_d_ in cattle slurry from barns is consumed over a collection period of 30 days [84], and the much longer retention time in Kariyapperuma [6]. The model inputs for the simulation are provided in S4 Appendix. Measured temperature and slurry mass data were linearly interpolated to provide daily values for simulations. The dataset consisted of seasonal campaigns with daily measurements of temperature and slurry mass during two years. Gap-filling to obtain a complete dataset covering 730 days was done by referring to corresponding data from a second measurement year (e.g., because temperature data was missing for spring 2010, we used measurements from spring 2011) (S4 Appendix). This extended dataset was used as model input, and then CH_4_ emission was simulated for five consecutive 730-day periods to ensure stable predictions. The fifth simulation round was compared to CH_4_ emissions measured by Kariyapperuma et al. [6].

## Results and discussion

### Model behavior

In this section effects of residual slurry and methanogen enrichment, temperature changes, and pH on predicted CH_4_ production are presented in order to show the behavior of the model.

#### Residual fraction and methanogen enrichment

**Fig 5:**
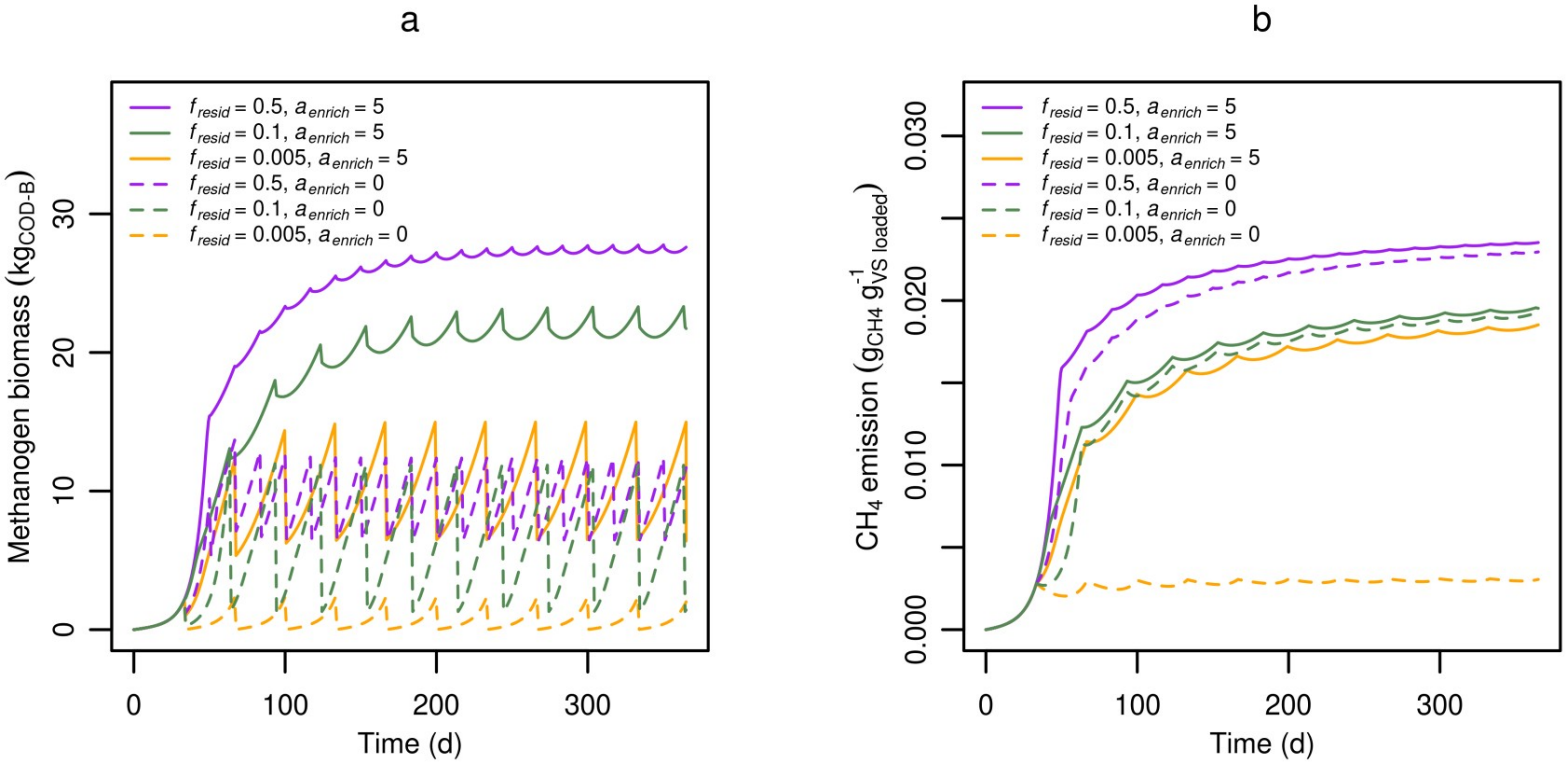
Predicted effects of residual slurry fraction on methanogens and methane production. **(a)** Total methanogen biomass and **(b)** Cumulative CH_4_ emission as affected by the residual fraction (*f*_*resid*_) of slurry after slurry removal assuming a high degree of microbial enrichment (*a*_*enrich*_), or no enrichment.

The residual fraction of slurry after export and the enrichment of active methanogens in the residue are expected to enhance methane production from fresh excreta. Fig 5 shows predicted effects of varying the residual fraction of slurry between 1 and 50% with or without enrichment of methanogens in the residue. Total methanogen biomass (Fig 5a) and CH_4_ production (Fig 5b) were correlated, and both quantities were substantially reduced when the slurry channel was emptied to 0.5% (*f*_*resid*_ = 0.005) as compared to 10% or 50% (*f*_*resid*_ = 0.1 or 0.5). The differences could be partially explained by the average amount of slurry in the tank, but was primarily a result of the smaller methanogen population being retained when the residual slurry fraction was low. Changing the microbial enrichment factor (*a*_*enrich*_) significantly impacted methanogen biomass only for the lowest residual fraction *f*_*resid*_ = 0.005. At *f*_*resid*_ = 0.5, and *f*_*enrich*_ = 5, methanogen retention was effectively close to 100%, resulting in complete consumption of available VFAs, and hydrolysis limiting CH_4_ production, which explains why CH_4_ production was almost identical for *f*_*enrich*_ = 5 and 0. Altogether, the results in Fig 5 demonstrate that a substantial CH_4_ reduction can be expected by near-complete emptying of the slurry channel.

#### Temperature effects

**Fig 6:**
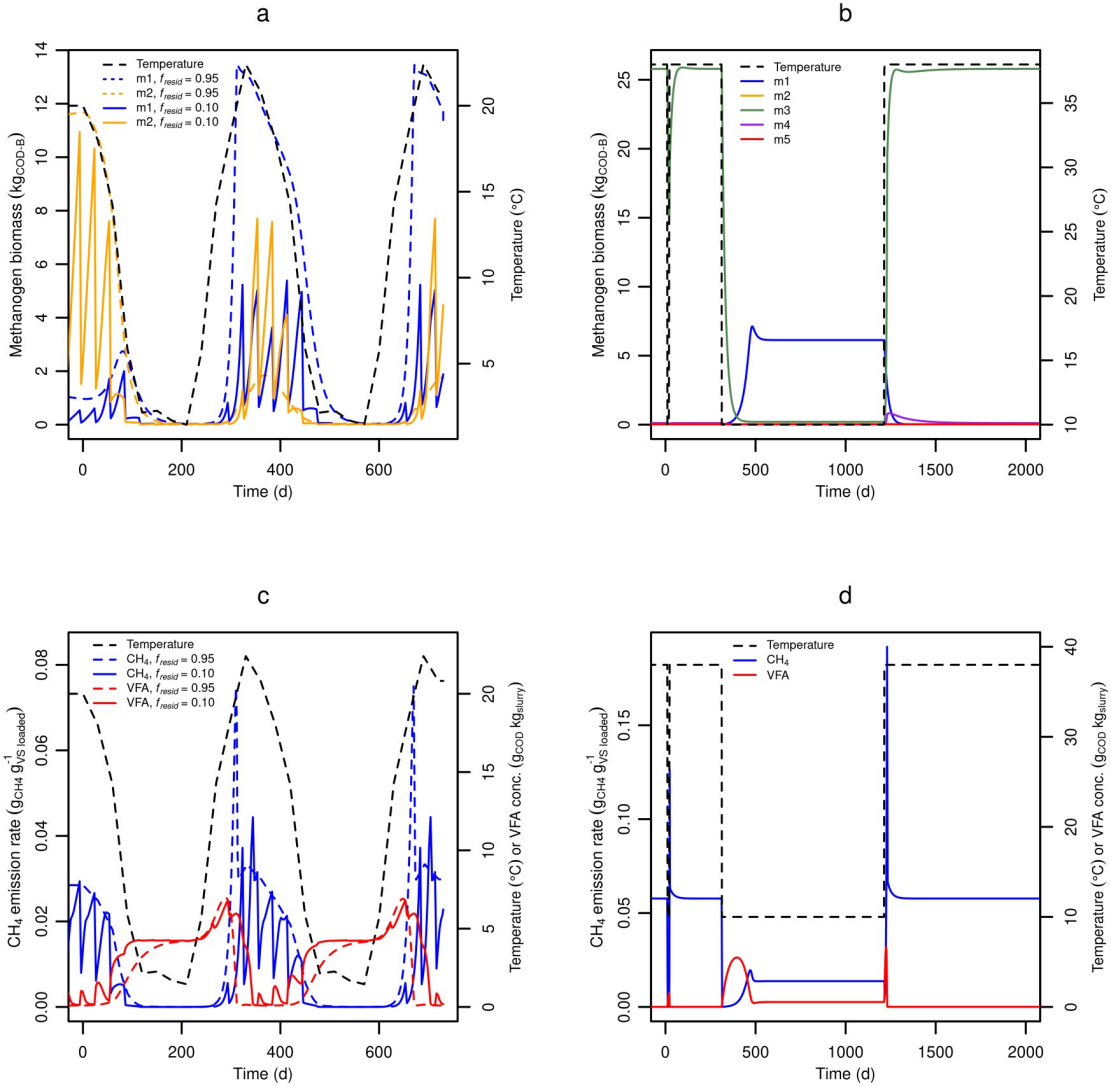
Predicted temperature effects on methanogens and methane production. **(a)** Methanogen biomass and **(c)** CH_4_ emission during gradual slurry temperature changes as predicted with a high and low residual slurry fraction in the storage (f_resid_). **(b)** Methanogen biomass and **(d)** CH_4_ emission during short- and long-term temperature changes using a large residual fraction of slurry (*f*_*resid*_) of 0.95.

The implementation of temperature sensitive methanogen groups allowed for simulating effects of gradual (seasonal) temperature changes, as well as transient and prolonged effects of rapid temperature change (i.e. slurry export from a barn to an outside storage, or from the animal tract to a slurry channel or pit). Fig 6a shows methanogen groups responding to a seasonal change in slurry temperature (temperatures from Kariyapperuma et al. [6]). It was predicted that at high residual slurry fraction (*f*_*resid*_ = 0.95) the methanogen biomass (Fig 6a) and CH_4_ emission (Fig 6c) will peak when the temperature peaks, but the response to the temperature change was not immediate neither with increasing nor decreasing temperature. The delay reflects the time required for a change in microbial biomass. On the other hand, significant hysteresis was observed when the residual fraction was low (*f*_*resid*_ = 0.1), with methanogen biomass and CH_4_ emission peaking 1–2 months after the temperature. A similar delay was seen in Kariyapperuma et al. [6], where relatively large portions of manure were added and removed, similar to the use of a small residual fraction. In Fig 6c, a large CH_4_ emission spike was predicted in response to increasing temperature, which reflects the rapid consumption of VFAs that had accumulated during the preceding cold period. This result reflects a low sensitivity of hydrolysis and fermentation to decreasing temperature in the model, compared to methanogenesis, although in reality this dynamic may be more complex. However, the accumulation of VFAs after rapid temperature changes has been observed in multiple studies [24, 27, 85]. In Fig 6b and 6d the effects of an instantaneous temperature decrease lasting 10 or 300 days are shown. Methanogen numbers declined only slightly during a 10-day temperature decrease (Fig 6b), resulting in a significant CH_4_ spike once the temperature was raised again (Fig 6d). In contrast, during a 900-day temperature decrease, m3 completely disappeared, resulting in a longer period of low CH_4_ production when the temperature later increased. Concomitantly, while a surplus of VFA substrate was transiently available after the temperature increase (Fig 6d), m4 grew alongside m3, but as VFAs were depleted, m4 was outcompeted again. In reality both m3 and m4 may need to acclimatize to the environment before consuming VFAs, which in the model could be accounted for by implementing a lag phase where CH_4_ production from a single methanogen population is temporarily halted in order to adjust cellular metabolism to new conditions, before resuming CH_4_ production. This mechanism, however, seems very difficult to describe with equations relating to actual biochemical processes due to complexity and lack of knowledge about the lag phase [86].

The new model effectively assumes a linear temperature dependency of long-term CH_4_ production rate (under non-limiting conditions), an assumption also made in other models [27, 51, 87]. However, for short-term dynamics of CH_4_ emissions there is solid evidence for an Arrhenius-like temperature dependency of hydrolysis and methanogenesis [22, 45, 58], and the long-term link between temperature and CH_4_ production rate remains to be studied systematically, particularly in the psychrophilic temperature range where slurry is often stored.

#### Acidification

**Fig 7:**
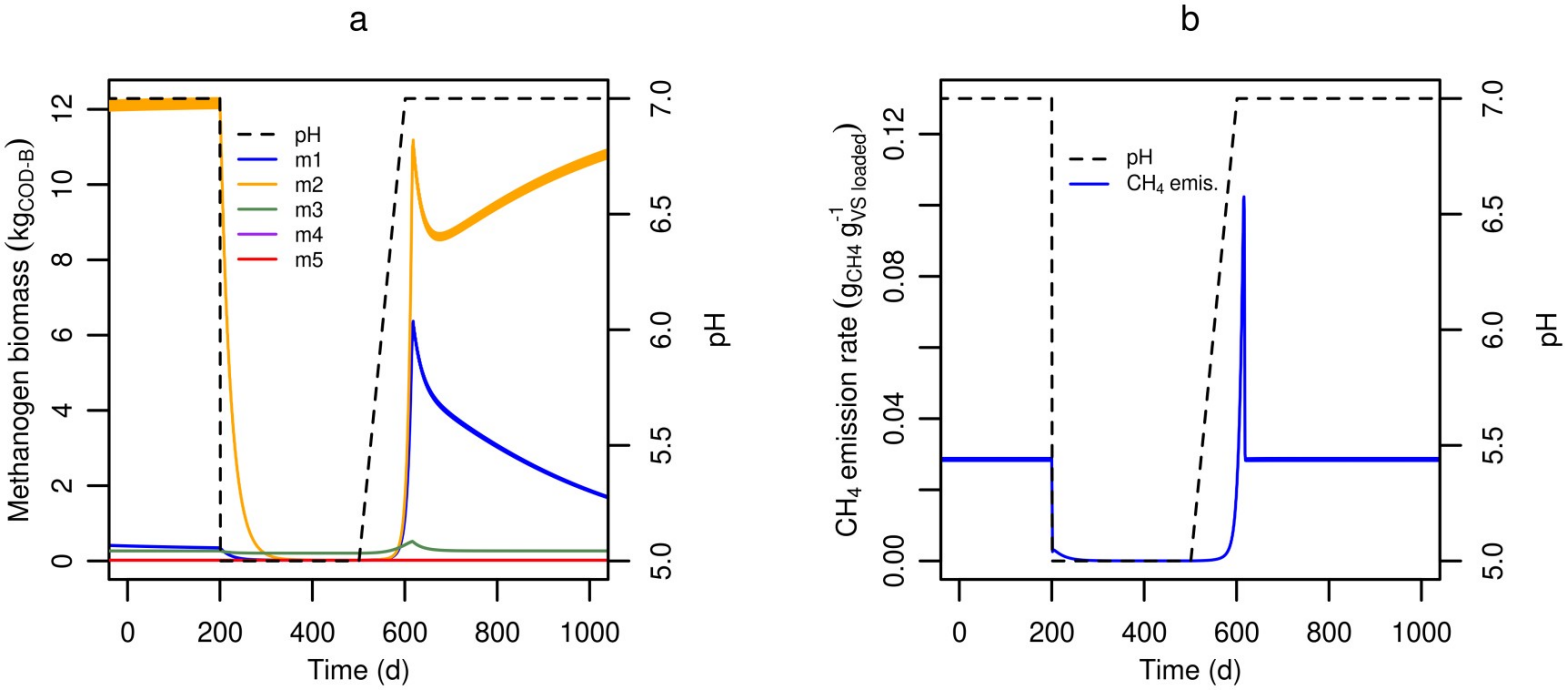
Predicted pH effects on methanogens and methane production. **(a)** Methanogen biomass and **(b)** CH_4_ emission responses to pH changes. The residual fraction of slurry (*f*_*resid*_) was set to 0.95.

Acidification to suppress ammonia volatilization has been shown to reduce CH_4_ emissions from cattle and pig slurry by 70–90% [15, 31, 37]. Fig 7 shows the predicted response to an instantaneous drop in pH, as in acidification in a storage tank. The immediate reduction in pH resulted in net microbial decay (Fig 7a) while immediately reducing CH_4_ emissions (Fig 7b). The apparent dominance of the m3 group during low pH was a consequence of its naturally higher abundance in the fresh slurry that was added each day. Once pH increased, the methanogens recovered and CH_4_ emissions rose again. However, in practical slurry management systems, the pH drop is typically achieved by sulfuric acid treatment, which inevitably raises the SO_4_^2-^ concentration to a level where sulfate reducing bacteria gain a thermodynamic advantage over methanogens. Therefore the model includes an optional sulfate reducer group (sr1) as demonstrated in S5 Appendix. Inclusion of sr1 decreased the magnitude and delayed by several months the CH_4_ peak after the pH was raised again. This response resulted primarily from increased competition between methanogen groups and sr1 for VFA substrate when SO_4_^2-^ was abundant, reflecting the known competition between the two groups [88]. A simulation with sr1 is probably more realistic for modelling acidification of slurry with sulfuric acid, but has the disadvantage of introducing additional parameters with associated uncertainty.

Similar to the effect of pH, the inhibiting effects of total ammoniacal nitrogen (TAN) and H_2_S were modelled by factoring a term directly onto the substrate utilization rate. An example of TAN inhibition is presented in S6 Appendix.

### Sensitivity analysis

**Fig 8:**
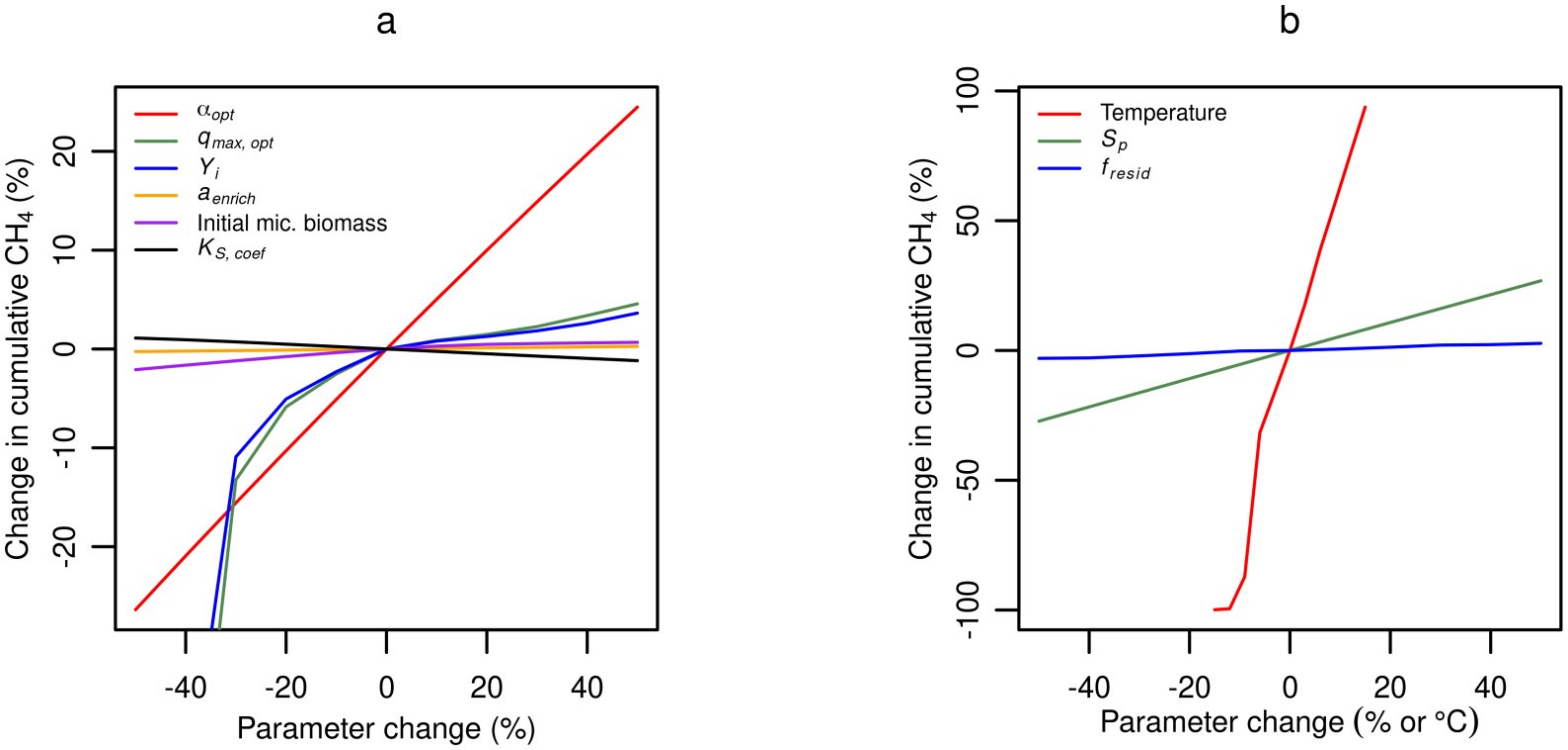
Model sensitivity to parameters and input variables. Model output sensitivities to **(a)** parameters and **(b)** input variables. Initial microbial biomass refers to changes in both the initial concentration of methanogens in the slurry inoculum and the fresh influent slurry. For parameter values, see S3 Appendix.

Cumulative CH_4_ production was most sensitive to the hydrolysis rate constant parameter, *α*_*opt*_, and the temperature input variable, *T* (Fig 8a and 8b). The model response is relatively insensitive to increases in the Monod parameters, but sensitive to decreases in the yield (*Y*_*i*_) and maximum substrate utilization rate (*q*_*max*,*opt*_). It is important to state that non-default parameters may significantly change model sensitivity to other parameters. Hydrolysis remains the least well-defined step in anaerobic digestion [43], and it can be considered the most critical input for the model performance. Significant effort should therefore be made to determine *α*_*opt*_.

### Model application

**Fig 9:**
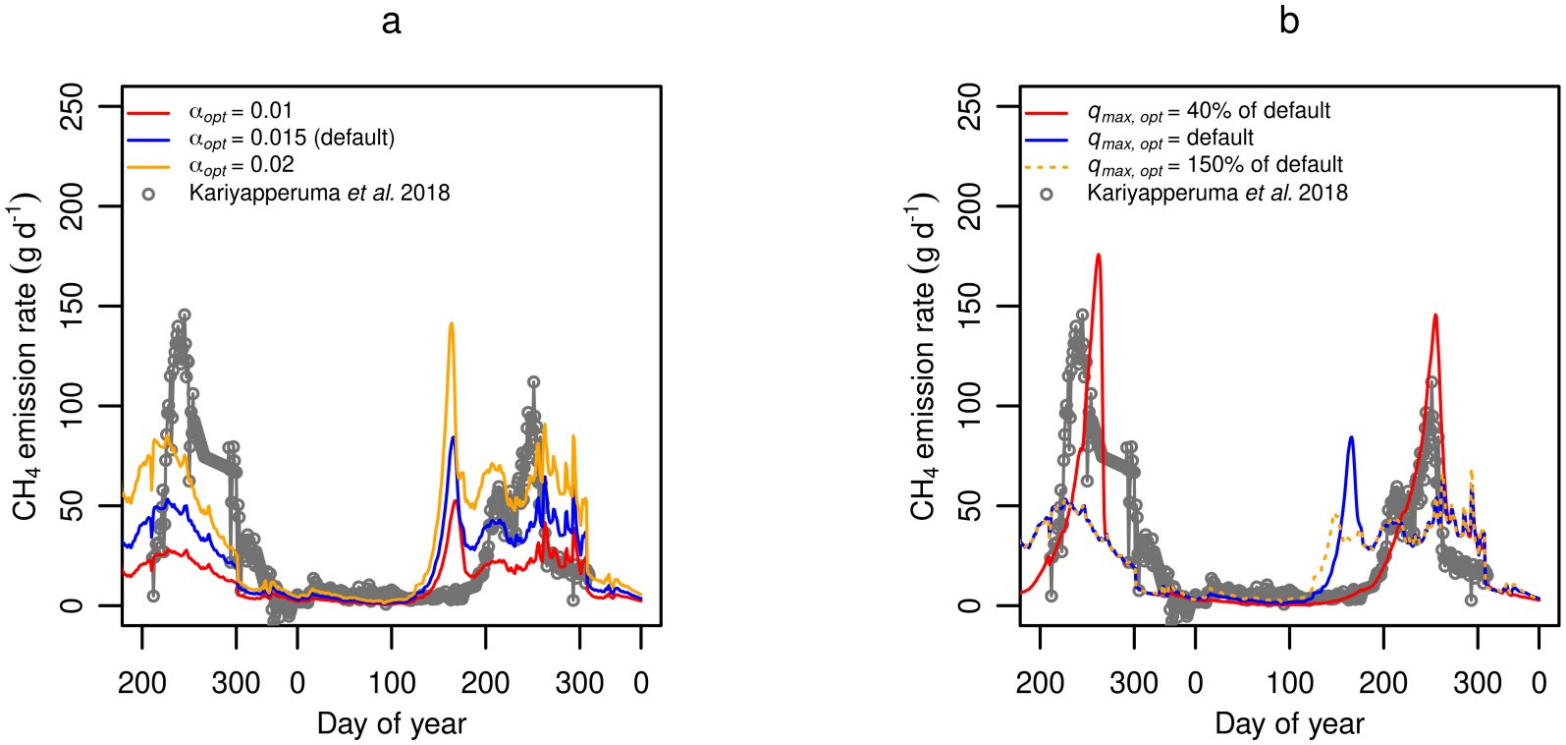
Model application to case study. Model results versus measured [6] CH_4_ emission from a full scale slurry tank with periodic slurry introduction applying different ***(a)*** hydrolysis rates (α_*opt*_) and ***(b)*** maximum substrate utilization rates at optimum temperature (*q*_*max*,*opt*_).

In Fig 9 the model was applied to the case study of Kariyapperuma et al. [6] to show the qualitative responses of the model against real measurements. The simulation was run with different hydrolysis rates (Fig 9a), which resulted in CH_4_ emission peaks of different magnitude, with *α*_*opt*_ = 0.02 matching best with measurements in terms of peak height. However, emissions were predicted to occur much too soon. The substrate utilization rate (*q*_*max*,*opt*_) was reduced, which postponed the CH_4_ emission peaks to match better with data (Fig 9b). As suggested in Fig 4b, methanogenesis is rate-limiting at low temperatures, explaining the observed effect of reducing *q*_*max*,*opt*_. There is a need for model validation in controlled experiments, and with the necessary input data. The total concentration and relative fractions of degradable and slowly degradable particulate material can be measured [82], but this is rarely done. Furthermore, emission studies on full-scale storages often report only the composition of slurry in the storage and not information about the influent slurry composition [6, 89]. The new model presented here relies on characterization of the influent slurry composition to determine hydrolysis rate and CH_4_ potential, and we stress therefore the importance of accurately measuring degradable particulate material and VFAs for accurate prediction of CH_4_ emission.

## Conclusions

With multiple groups (populations) of methanogens, a mechanistic model of methane production from animal manure or similar wastes can reproduce complex observed responses to temperature, in particular, the distinctly different short- and long-term responses to temperature change. The new model described here, implemented in the ABM R package, is a flexible tool that can facilitate research on CH_4_ emission and its control. Accounting for temporary inactivation of methanogens, methane oxidation, and possibly other processes may be necessary for the most accurate predictions, and model extension is possible. An application of the model to field data showed that detailed measurements of slurry organic matter composition will be needed for model extension and future application at all scales.

## Supporting information

S1 Appendix Assumptions about unit conversion and manure composition. Explanation and numeric values for VS composition and conversion between VS and COD.
(PDF)

S2 Appendix Additional model equations. Additional equations used in the model not described in paper.
(PDF)

S3 Appendix Parameters and variables. Parameter and variable values used as defaults and in “Model behavior” section.
(PDF)

S4 Appendix Input data. Input data used for simulating the conditions in the case study by Kariyapperuma et al. [6].
(PDF)

S5 Appendix Sulfate reducers and low pH. Model predictions for the effect of acidification with inclusion of a sulfate reducer population.
(PDF)

S6 Appendix Ammonia inhibition dynamics. Model predictions for ammonia inhibition of methanogens.
(PDF)
