# Supplementary material for: A mechanistic model of methane emission from animal slurry with a focus on microbial groups
Source: PLoS One. 2021 Jun 10;16(6):e0252881. doi: 10.1371/journal.pone.0252881 (PMC8191904; doi:10.1371/journal.pone.0252881)
Supplement: S1 Appendix — Explanation and numeric values for VS composition and conversion between VS and COD. (PDF) [file pone.0252881.s001.pdf]

S1 Appendix. Assumptions about unit conversion and manure composition

The conversion of VS to COD is based on the algorithm described by Rittmann and McCarty [1] using the biogas R package v. 1.23 [2]. The calculated oxygen demand (COD') per weight (g) of a molecule with formula $C_nH_aO_bN_c$ is given by equation S1:

$$\frac{COD'}{weight} = \frac{(2\cdot n+0.5\cdot a-1.5\cdot c-b)\cdot 16}{12\cdot n+a\cdot 16\cdot b+14\cdot c} \quad \text{(S1)}$$

The default influent slurry composition is based on the composition of cattle manure from [3] and the COD:VS ratio can be estimated as a weighted average based on COD' (Table S1). However, degradable particulate material S_p includes only the weighted average of components considered degradable (which here is considered proteins, lipids and degradable carbohydrates). Further, VFA is defined separately from other COD sources in the influent slurry. Hence, the COD:VS ratio of S_p and VFA is also given in Table S1.

Table S1. Assumptions about unit conversion and manure composition. Composition of fresh pig and cattle manure and their respective COD:VS ratio of total organic matter (OM), degradable particulate material (Sp) ratios, and COD:VSd ratios.

			Pig manure		Cattle manure	
Organic matter (g_{VS} kg_{Slurry}^{-1})			80		110	
Component	Formula	g_{COD} g^{-1}	g g_{VS}^{-1}	g_{COD} g_{VS}^{-1}	g g_{VS}^{-1}	g_{COD} g_{VS}^{-1}
VFA	$C_2H_4O_2$	1.07	0.08	0.09	0.04	0.04
Protein	$C_5H_7O_2N$	1.42	0.18	0.25	0.15	0.21
Lipids	$C_{57}H_{104}O_6$	2.89	0.12	0.35	0.07	0.20
Degradable carbohydrate	$C_6H_{10}O_5$	1.18	0.39	0.47	0.43	0.51
Non-degradable carbohydrate	$C_6H_{10}O_5$	1.18	0.18	0.21	0.19	0.23
Lignin	$C_{10}H_{13}O_3$	2.08	0.05	0.10	0.12	0.25
**Organic matter (OM)**			**1.00**	**1.47**	**1.00**	**1.44**
		g_{COD} g^{-1}	g g_{VSd}^{-1}	g_{COD} g_{VSd}^{-1}	g g_{VSd}^{-1}	g_{COD} g_{VSd}^{-1}
Protein	$C_5H_7O_2N$	1.42	0.26	0.37	0.23	0.33
Lipids	$C_{57}H_{104}O_6$	2.89	0.17	0.50	0.11	0.30
Degradable carbohydrate	$C_6H_{10}O_5$	1.18	0.57	0.67	0.66	0.79
**Degradable particulate material (S_p)**			**1.00**	**1.54**	**1.00**	**1.42**

The default model input concentrations of VFA, S_p and OM were calculated according to equation S2-S4. For estimating $C_{Sp,in}$ the VSd:VS ratio was calculated as the mean from B0:Bu measurements [3,4] yielding 0.42 g_{VSd} g_{VS}^{-1}.

$$C_{OM,in} = g_{COD}g_{VS}^{-1} \cdot g_{VS}kg_{Slurry}^{-1} = 1.44 \cdot 110 = 158.4\ g_{COD}kg_{Slurry}^{-1} \quad \text{(S2)}$$

$$C_{Sp,in} = g_{COD}g_{VSd}^{-1} \cdot g_{VS}kg_{Slurry}^{-1} \cdot g_{VSd}g_{VS}^{-1} = 1.42 \cdot 110 \cdot 0.42 = 65.6\ g_{COD}kg_{Slurry}^{-1} \quad \text{(S3)}$$

$$C_{VFA,in} = g_{COD}g_{VS}^{-1} \cdot g_{VS}kg_{Slurry}^{-1} \cdot g_{VFA}kg_{Slurry}^{-1} = 1.07 \cdot 110 \cdot 0.04 = 4.5\ g_{COD}kg_{Slurry}^{-1} \quad \text{(S4)}$$

Coefficients for CO_2 emission from anaerobic reactions (including all inorganic C produced) were based on the following stoichiometric equations, which were calculated as described in Rittmann and McCarty (2001) using the predBg() function in the biogas package [2]. The net fraction of substrate used for cell synthesis (f_s) was fixed at 0.03. A formula of $C_5H_7O_2N$ was used for cell biomass [1].

Pig manure:

$$C_{13.2}H_{22.2}O_{6.5}N + 5.8H_2O \rightarrow 7.15CH_4 + 4.69CO_2 + 0.089C_5H_7O_2N + 0.91NH_4^+ + 0.91HCO_3^-$$

Cattle manure:

$$C_{16.6}H_{27.8}O_{10}N + 6.0\ H_2O \rightarrow 8.6CH_4 + 6.54CO_2 + 0.11C_5H_7O_2N + 0.89NH_4^+ + 0.89HCO_3^-$$

For aerobic oxidation and sulfate reduction, CO_2 production was calculated from the C content of the substrate (using the empirical chemical formulas given above), reduced by f_s values of 0.1 and 0.01, respectively [1].

References

1. Rittmann BE, McCarty PL. Environmental Biotechnology: Principles and Applications. Boston: McGraw-Hill; 2001.

2. Hafner SD, Koch K, Carrere H, Astals S, Weinrich S, Rennuit C. Software for biogas research: Tools for measurement and prediction of methane production. SoftwareX. 2018;7: 205–210. doi:10.1016/j.softx.2018.06.005

3. Møller HB, Sommer SG, Ahring BK. Methane productivity of manure, straw and solid fractions of manure. Biomass and Bioenergy. 2004;26: 485–495. doi:10.1016/j.biombioe.2003.08.008

4. Møller HB, Sommer SG, Ahring BK. Biological degradation and greenhouse gas emissions during pre-storage of liquid animal manure. Journal of Environmental Quality. 2004;33: 27–36. doi:10.2134/jeq2004.2700
