# Supplementary material for: A mechanistic model of methane emission from animal slurry with a focus on microbial groups
Source: PLoS One. 2021 Jun 10;16(6):e0252881. doi: 10.1371/journal.pone.0252881 (PMC8191904; doi:10.1371/journal.pone.0252881)
Supplement: S2 Appendix — Additional equations used in the model not described in paper. (PDF) [file pone.0252881.s002.pdf]

S2 Appendix. Additional model equations

Table S2. Additional equations (and default constants) used in the model. Sources given only where source is not described in paper or otherwise obvious.

Symbol	Expression	Unit	Description	Source
**Chemical speciation**				
$C_{TAN,initial}$	$C_{TAN,in}$	$g_N\ kg_{slurry}^{-1}$	Concentration of total ammoniacal nitrogen	
C_{NH_3}	$\frac{1}{1+10^{(-log_{10}(\gamma_{NH_4} \cdot K_{a,NH_3})-pH)}} \cdot C_{TAN,in}$	$g_N\ kg_{slurry}^{-1}$	Concentration of ammonia	[1]
$C_{NH_4^+}$	$C_{TAN,in} - C_{NH_3}$	$g_N\ kg_{slurry}^{-1}$	Concentration of ammonium	
C_{H_2S}	$1 - \frac{1}{1+10^{(-log_{10}K_{a,H_2S}-pH)}} \cdot C_{sulfide}$	$g_{sulfur}\ kg_{slurry}^{-1}$	Concentration of hydrogen sulfide	
$log_{10}\ K_{a,NH_3}$ [a]	$0.09046 - \frac{2729.31}{T}$	-	Acid dissociation constant of ammonia	[2]
$log_{10}\ K_{a,H_2S}$ [a]	$-7.051 + e^{0.029 \cdot (T-298)}$	-	Acid dissociation constant of hydrogen sulfide	
**Biological kinetics**				
q_{max}	$\frac{q_{max,opt} \cdot (T-T_{max}) \cdot (T-T_{min})^2}{(T_{opt}-T_{min}) \cdot [(T_{opt}-T_{min}) \cdot (T-T_{opt}) - (T_{opt}-T_{max}) \cdot (T_{opt}+T_{min}-2 \cdot T)]}$	$g_{COD-S}\ g_{COD-B}^{-1}\ d^{-1}$	Maximum substrate utilization rate	
α	$\frac{\alpha_{opt} \cdot (T-T_{\alpha,max}) \cdot (T-T_{\alpha,min})^2}{(T_{\alpha,opt}-T_{\alpha,min}) \cdot [(T_{\alpha,opt}-T_{\alpha,min}) \cdot (T-T_{\alpha,opt}) - (T_{\alpha,opt}-T_{\alpha,max}) \cdot (T_{\alpha,opt}+T_{\alpha,min}-2 \cdot T)]}$	d^{-1}	Maximum hydrolysis rate	
K_S [b]	$K_{S,coef} \cdot 0.8157 \cdot e^{(-0.063 \cdot T)}$	$g_{COD-S}\ kg_{Slurry}^{-1}$	Half max saturation constant	
**Mass transfer**				
k_{H,O_2} [a]	$41.6 \cdot e^{1700 \cdot (\frac{1}{T}-\frac{1}{298})}$	$g_{O_2}\ m^{-3}\ atm^{-1}$	Henry's law constant for oxygen (liquid:gas)	[3]
k_{L,O_2}	0.5	$m\ d^{-1}$	Overall (liquid unit) mass transfer coefficient for oxygen	
k_{L,H_2S}	0.02	$m\ d^{-1}$	Overall (liquid unit) mass transfer coefficient for hydrogen sulfide	
r_{O_2}	$k_{L,O_2} \cdot A \cdot (0.208 k_{H,O_2} - 0)$	$g_{COD}\ d^{-1}$	Aerobic respiration rate (assumed to be mass transfer limited)	
j_{H_2S}	$k_{L,H_2S} \cdot A \cdot (C_{H_2S} - 0)$	$g_{sulfur}\ d^{-1}$	H_2S emission rate	
**Management**				
$f_{resid,Xi}$	$\frac{e^{ln(\frac{1}{f_{resid}-1})+a_{enrich}}}{1+e^{ln(\frac{1}{f_{resid}-1})+a_{enrich}}}$	-	Fraction of biomass retained after emptying	
X_i	$if\ M_m \geq\ M_{m,max}, X_i = X_i \cdot f_{resid,Xi}$	g_{COD-B}	biomass retained after emptying	
S_i	$if\ M_m \geq\ M_{m,max}, S_i = S_i \cdot f_{resid}$	g_{COD-S}, $g_{H2S-sulfur}$, $g_{SO4-sulfur}$	components retained after emptying	
M_m	$if\ M_m \geq\ M_{m,max}, M_m = M_m \cdot f_{resid}$	kg_{Slurry}	slurry retained after emptying	
[a] T in Kelvin, [b] T in °C				

References

1. Hafner SD, Bisogni JJ. Modeling of ammonia speciation in anaerobic digesters. Water Research. 2009;43: 4105–4114. doi:10.1016/j.watres.2009.05.044

2. Clegg SL, Whitfield M. A chemical-model of seawater including dissolved ammonia and the stoichiometric dissociation-constant of ammonia in estuarine water and seawater from -2C to 40C. Geochimica et Cosmochimica Acta. 1995;59: 2403–2421.

3. Sander R. Henry's Law Constants. In: Linstrom PG, Mallard WG, editors. NIST Chemistry WebBook, NIST Standard Reference Database Number 69. Gaithersburg, MD: National Institute of Standards and Technology; 2017. Available: http://webbook.nist.gov
