# Supplementary material for: A mechanistic model of methane emission from animal slurry with a focus on microbial groups
Source: PLoS One. 2021 Jun 10;16(6):e0252881. doi: 10.1371/journal.pone.0252881 (PMC8191904; doi:10.1371/journal.pone.0252881)
Supplement: S3 Appendix — Parameter and variable values used as defaults and in “Model behavior” section. (PDF) [file pone.0252881.s003.pdf]

S3 Appendix. Parameters and variables

Table S3. Parameters and variables. Default values of parameters/variables (black) and values used for presenting model behavior dynamics (red).

Symbol	Default value	Unit	Description
F_{in}	1000<br><br>Figure 3a = 0; Figure 3b = 1/50; Figure 9 = measurements used instead;	$kg_{Slurry}\ d^{-1}$	Fresh slurry production
f_{resid}	0.1;<br><br>Figure 3a = 0; Figure 3b = 0.95; Figure 4b = 0.95; Figure 5 = 0.5, 0.1, and 0.001; Figure 6a,c = 0.1 and 0.95; Figure 6b,d = 0.95; Figure 7 = 0.95; Figure 8 = 0.5 and variable; Figure 9 = measurements used instead;		Fraction of slurry retained after emptying
A	11;<br><br>Figure 3a = 0, Figure 9 = 730	m^2	Exposed surface area of slurry
$M_{m,max}$	33333<br><br>Figure 3 = 1, Figure 9 = measured data used instead;	kg	Maximum capacity of tank or channel
T	20;<br><br>Figure 3a = 5 to 52 by 0.5, Figure 3b = 18, 37, and 52; Figure 4b = 0-60 °C with gradual temperature increment of 0.006 °C per day; Figure 6a,c = gradual changes between 0 and 24 °C; 6b,d = instant changes from 10 to 38 °C; Figure 8 = 20 ± 15 °C; Figure 9 = measurements used instead	°C	Slurry temperature
pH	7; Figure 7 = 7 then change to 5 over 1 day and later back to 7 over 100 days.	pH	Slurry pH
$C_{OM,in}$	160;	$g_{COD}\ kg_{Slurry}^{-1}$	Concentration of organic matter in the fresh slurry
$C_{Sp,in}$	65;<br><br>Figure 8 = 65 ± 50%	$g_{COD\text{-}S}\ kg_{Slurry}^{-1}$	Degradable particulate material in the fresh slurry
$C_{VFA,in}$	4.2;	$g_{COD\text{-}S}\ kg_{Slurry}^{-1}$	Volatile fatty acids in the fresh slurry
$C_{SO4,in}$	0.2;	$g_{SO4\text{-}sulfur}\ kg_{Slurry}^{-1}$	Sulfur as sulfate in the fresh slurry
$C_{sulfide,in}$	0.0;	$g_{H2S\text{-}sulfur}\ kg_{Slurry}^{-1}$	Sulfur as sulfide in the fresh slurry
$C_{TAN,in}$	1;	$g_{TAN\text{-}N}\ kg_{Slurry}^{-1}$	Total ammonia nitrogen in the fresh slurry
$C_{Xi,in}$	m1 = 0.01; m2 = 0.005; m3 = 0.005; m4 = 0.005; m5 = 0.001; for optional sr1 = 0.001<br><br>Figure 8 = (m1 = 0.01; m2 = 0.005; m3 = 0.005; m4 = 0.005; m5 = 0.001;) ± 50%;	$g_{COD\text{-}B}\ kg_{Slurry}^{-1}$	Microbial populations in fresh slurry
$C_{OM,initial}$	160;	$g_{COD}\ kg_{Slurry}^{-1}$	Initial concentration of organic matter
$C_{Sp,initial}$	65;<br><br>Figure 8 = 65 ± 50%;	$g_{COD\text{-}S}\ kg_{Slurry}^{-1}$	Initial concentration of degradable particulate material in the slurry
$C_{VFA,initial}$	4.2;	$g_{COD\text{-}S}\ kg_{Slurry}^{-1}$	Initial concentration of VFA in the slurry
$C_{SO4,initial}$	0.2;	$g_{SO4\text{-}sulfur}\ kg_{Slurry}^{-1}$	Initial concentration of SO4-sulfur in the slurry
$C_{sulfide,initial}$	0.0;	$g_{sulfur}\ kg_{slurry}^{-1}$	Initial concentration of sulfide (S^{-2}+HS^{-}+H_2S)
$C_{Xi,initial}$	All groups = 0.001, but m3 = 0.01<br><br>Figure 8 = (All groups = 0.001, but m3 = 0.01) ± 50%;	$g_{COD\text{-}B}\ kg_{Slurry}^{-1}$	Initial concentration of microbial populations in the slurry
k_d	All groups = 0.02	d^{-1}	Death and decay rates

$K_{S,coef}$	All groups = 1, for optional sr1 = 0.4	$g_{COD\text{-}S}\ kg_{Slurry}^{-1}$	coefficient for the half max substrate concentration constant
$K_{S,SO4}$	0.0067	$g_{SO4\text{-}sulfur}\ kg_{Slurry}^{-1}$	half max SO_4 concentration constant
a_{enrich}	All groups = 0;<br><br>Figure 5: all groups set to 0 or 5; Figure 8: 1 ± 50%;		Enrichment factor
Y_i	All methanogen groups = 0.05; for optional sr1 = 0.065<br><br>Figure 8: 0.05 ± 50%; Figure S5 (S5 Appendix): 0.04; Figure S6 (S6 Appendix): 0.04	$g_{COD\text{-}B}\ g_{COD\text{-}S}^{-1}$	Growth yield
$q_{max,opt}$	m1 = 3.6; m2 = 5.6; m3 = 7.2; m4 = 8; m5 = 8; for optional sr1 = 8;<br><br>Figure 8: (m1 = 3.6; m2 = 5.6; m3 = 7.2; m4 = 8; m5 = 8) ± 50%;<br><br>Figure 9: 50%, 75% and 100% of (m1 = 3.6; m2 = 5.6; m3 = 7.2; m4 = 8; m5 = 8)	$g_{COD\text{-}S}\ g_{COD\text{-}B}^{-1}\ d^{-1}$	Specific max substrate utilization rate at optimum temperature
T_{opt}	m1 = 18; m2 = 28; m3 = 36; m4 = 44; m5 = 55, for optional sr1 = 40	°C	Optimum temperature
T_{min}	m1 = 0; m2 = 8; m3 = 15; m4 = 26; m5 = 30, for optional sr1 = 5<br><br>Figure 3: red m3 only, blue m3 only = 0	°C	Min temperature of substrate utilization
T_{max}	m1 = 25; m2 = 38; m3 = 45; m4 = 51; m5 = 60, for optional sr1 = 50<br><br>Figure 3: red m3 only, blue m3 only = 55	°C	Max temperature of substrate utilization
α_{opt}	0.02<br><br>Figure 8: 0.015 ± 50%; Figure 9: 0.01, 0.015, and 0.02	d^{-1}	First order hydrolysis rate at optimum temperature
$T_{\alpha,opt}$	55	°C	Optimum temperature for hydrolysis
$T_{\alpha,min}$	0	°C	Min temperature for hydrolysis
$T_{\alpha,max}$	60	°C	Min temperature for hydrolysis
$KI_{NH3,min}$	All groups = 0.015	$g_{NH3\text{-}N}\ kg_{Slurry}^{-1}$	Min NH_3 inhibition constant
$KI_{NH3,max}$	All groups = 0.131	$g_{NH3\text{-}N}\ kg_{Slurry}^{-1}$	Max NH_3 inhibition constant
$KI_{NH4,min}$	All groups = 2.714	$g_{NH4+\text{-}N}\ kg_{Slurry}^{-1}$	Min NH_4 inhibition constant
$KI_{NH4,max}$	All groups = 4.764	$g_{NH4+\text{-}N}\ kg_{Slurry}^{-1}$	Max NH_4 inhibition constant
pH_{UL}	All groups = 8	pH	Upper pH inhibition constant
pH_{LL}	All groups = 6.5, for optional sr1 = 5.5	pH	Lower pH inhibition constant
KI_{H2S}	All groups = 0.23, for optional sr1 = 0.25	$g_{H2S\text{-}sulfur}\ kg_{Slurry}^{-1}$	H_2S inhibition constant
P_{CH4}	0.2507	$g_{CH4}\ g_{COD\text{-}S}^{-1}$	CH_4 productivity coefficient
$P_{CO2,anaerobic}$	0.53	$g_{CO2}\ g_{COD\text{-}S}^{-1}$	Anaerobic CO_2 productivity coefficient
$P_{CO2,aerobic}$	1.1	$g_{CO2}\ g_{COD\text{-}S}^{-1}$	Aerobic CO_2 productivity coefficient
$P_{CO2,sr}$	1.2	$g_{CO2}\ g_{COD\text{-}S}^{-1}$	Sulfate reducer CO_2 productivity coefficient
kL_{O2}	0.5	$m\ d^{-1}$	Mass transfer coefficient for O_2
kL_{H2S}	0.02	$m\ d^{-1}$	Mass transfer coefficient for H_2S
$f_{COD\text{-}S,Sulfur}$	0.5015	$g_{Sulfur}\ g_{COD\text{-}S}^{-1}$	Unit conversion factor
