# Supplementary material for: A mechanistic model of methane emission from animal slurry with a focus on microbial groups
Source: PLoS One. 2021 Jun 10;16(6):e0252881. doi: 10.1371/journal.pone.0252881 (PMC8191904; doi:10.1371/journal.pone.0252881)
Supplement: S4 Appendix — Input data used for simulating the conditions in the case study by Kariyapperuma et al. [6]. (PDF) [file pone.0252881.s004.pdf]

S4 Appendix. Input data

Table S4. Input data. Input data used for simulating the conditions in the case study of Kariyapperuma et al. (1).

**Influent/ initial slurry composition**	**Value**
$C_{Sulfide,in}$ ($g_{H2S\text{-}sulfur}$ kg_{Slurry}^{-1})	0.0
$C_{SO4,in}$ ($g_{SO4\text{-}sulfur}$ kg_{Slurry}^{-1})	0.2
$C_{TAN,in}$ ($g_{TAN\text{-}N}$ kg_{Slurry}^{-1})	1.091
$C_{Sp,in}$ ($g_{COD\text{-}S}$ kg_{Slurry}^{-1})	40.98
$C_{VFA,in}$ ($g_{COD\text{-}S}$ kg_{Slurry}^{-1})	1.4
$C_{OM,in}$ (g_{COD} kg_{Slurry}^{-1})	103.93
**Other input variables**	
pH	7.33
A (m^2)	730
Temp (°C)	Variable (See Fig. S4 below)
Slurry mass (kg_{Slurry})	Variable (See Fig. S4 below)

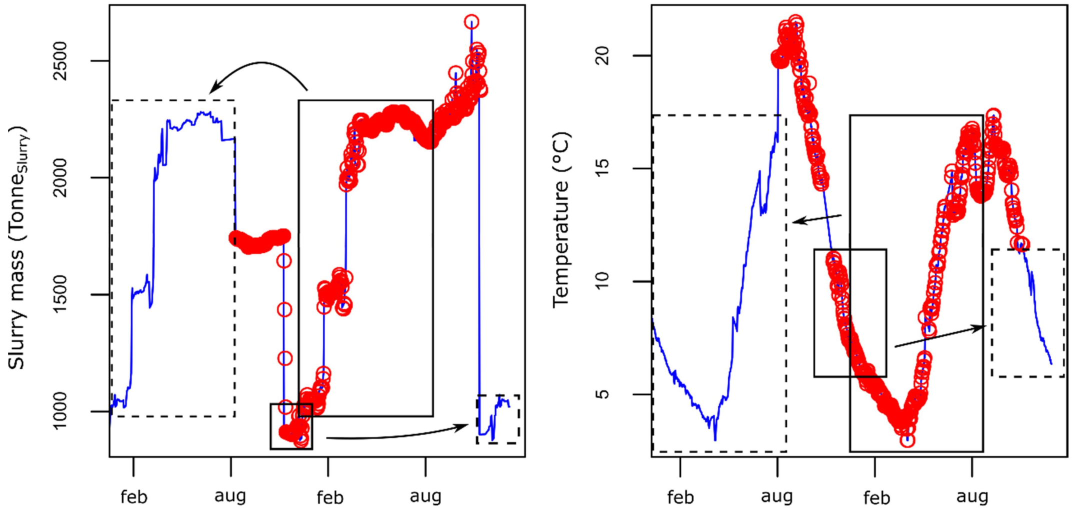

Figure S4. Data extension and variable input data. (a) Measured slurry mass (red circles) and model input mass (blue line) and **(b)** measured slurry temperature (red circles) and model input temperatures (blue line) used for the simulation of the case study (1).

References

1. Kariyapperuma KA, Johannesson G, Maldaner L, VanderZaag A, Gordon R, Wagner-Riddle

C. Year-round methane emissions from liquid dairy manure in a cold climate reveal hysteretic pattern. Agric For Meteorol [Internet]. 2018;258(July 2017):56–65. Available from: https://doi.org/10.1016/j.agrformet.2017.12.185
