# Supplementary material for: A mechanistic model of methane emission from animal slurry with a focus on microbial groups
Source: PLoS One. 2021 Jun 10;16(6):e0252881. doi: 10.1371/journal.pone.0252881 (PMC8191904; doi:10.1371/journal.pone.0252881)
Supplement: S5 Appendix — Model predictions for the effect of acidification with inclusion of a sulfate reducer population. (PDF) [file pone.0252881.s005.pdf]

S5 Appendix. Sulfate reducers and low pH

In Fig. S5 the pH was reduced while simultaneously increasing the inlet slurry SO_4^{2-} concentration in raw slurry to 5.5 $g_{SO4\text{-}sulfur}$ kg_{Slurry}^{-1} corresponding to sulfuric acid treatment measurements [1,2]. During acidic conditions the effect of SO_4^{2-} was almost negligible, but once pH increased accumulated SO_4^{2-} and VFA was consumed by sulfate reducers (*sr1*), delaying the subsequent growth of *m2* (S5 Fig a) when comparing to Figure 7a in the paper. In S3 Fig b it is shown how the CH_4 peak after pH was increased is delayed significantly due to the initial dominance of sr1 (S5 Fig a). It was previously shown that SO_4^{2-} addition alone inhibited methanogenic activity and it was hypothesized that it was caused by H_2S inhibition [3]. However, using default inhibition constants, our model suggested that H_2S inhibition was insignificant in this scenario.

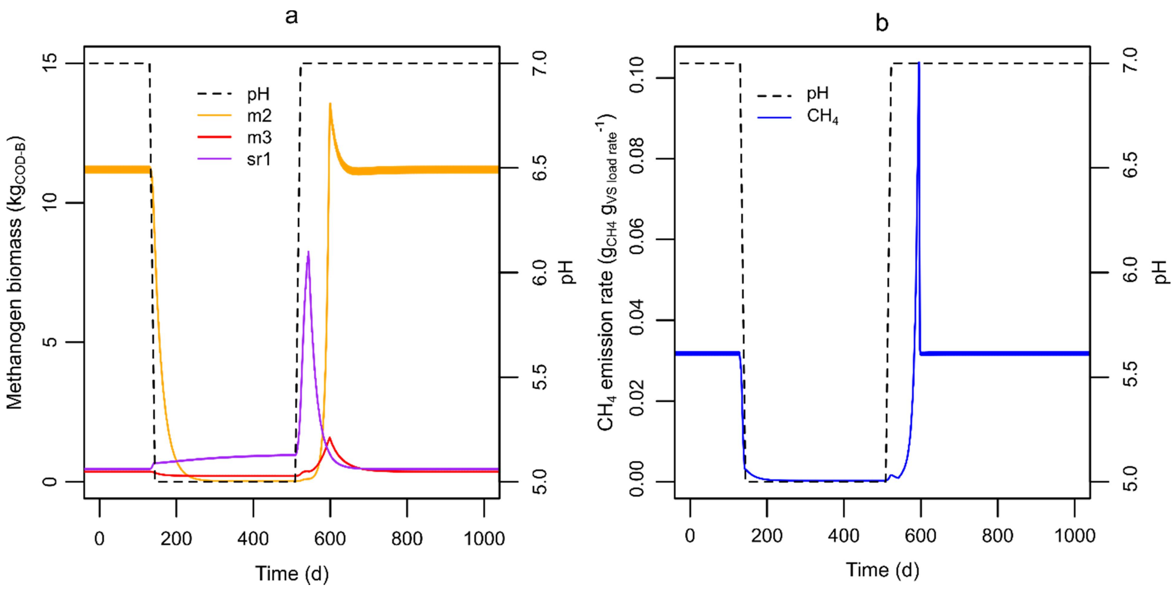

Fig S5. Sulfate reducers and low pH. (a) Methanogen and sulfate reducer (*sr1*) biomass and **(b)** corresponding CH4 emission. For *sr1* Yi = 0.065, $q_{max,opt}$ = 8, *T_opt* = 40 °C, *T_min* = 0 °C, *T_max* = 50 °C, and *pH_lwr* = 5.5. Other microbial parameters for *sr1* were similar to those of methanogen groups. The residual fraction of slurry (f_{resid}) was set to 0.95 for this simulation.

References

1. Dalby FR, Nikolausz M, Hansen MJ, Feilberg A. Sulfur transformations and methanogenic pathways in tannic acid-sodium fluoride inhibited and acidified livestock-and sludge waste. Applied Microbiology and Biotechnology. 2020;Submitted.

2. Eriksen J, Sørensen P, Elsgaard L. The Fate of Sulfate in Acidified Pig Slurry during Storage and Following Application to Cropped Soil. Journal of Environmental Quality. 2008;37: 280–286. doi:10.2134/jeq2007.0317

3. Petersen SO, Andersen AJ, Eriksen J. Effects of Cattle Slurry Acidification on Ammonia and Methane Evolution during Storage. Journal of Environment Quality. 2012;41: 88. doi:10.2134/jeq2011.0184
