# Supplementary material for: A mechanistic model of methane emission from animal slurry with a focus on microbial groups
Source: PLoS One. 2021 Jun 10;16(6):e0252881. doi: 10.1371/journal.pone.0252881 (PMC8191904; doi:10.1371/journal.pone.0252881)
Supplement: S6 Appendix — Model predictions for ammonia inhibition of methanogens. (PDF) [file pone.0252881.s006.pdf]

S6 Appendix. Ammonia inhibition dynamics

In Fig. S6 the effect of increasing the total ammoniacal nitrogen concentration (TAN) is seen for two methanogen groups that exhibit different sensitivity to TAN. While *m2* dominates at lower TAN concentrations the dominance flips around resulting in *m3* dominance at higher TAN concentrations. Eventually at even higher TAN concentrations *m3* is also significantly inhibited. Additionally, the model predicts a short drop followed by a long term increase in CH_4 emission when increasing TAN from 2.5 g_{TAN-N} kg_{Slurry}^{-1} to 3 g_{TAN-N} kg_{Slurry}^{-1}. These simulations are of particular interest in digester simulations where a nitrogen rich substrate might suddenly be fed to the reactor, disturbing the methanogenic community structure. Hydrogenotrophic methanogens are generally more tolerant to higher TAN levels than acetoclastic (1) , which can be captured with this flexible model by adjusting methanogen group parameters accordingly.

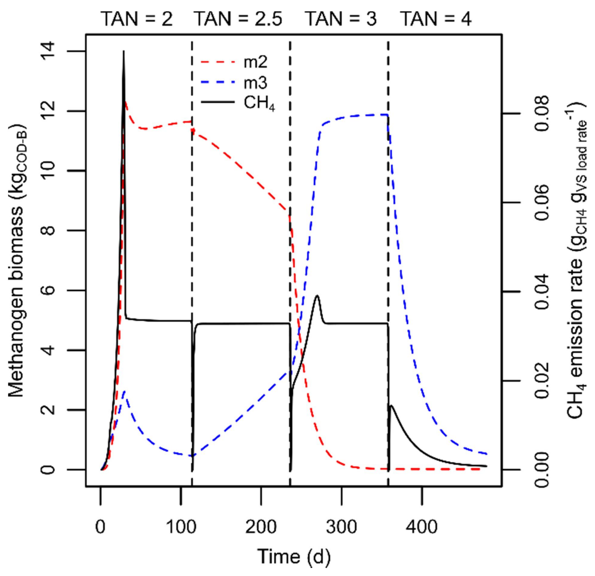

Fig S6. Total ammonia nitrogen inhibition dynamics. TAN inhibition of methanogens with different sensitivity to TAN. For m2 the TAN inhibition parameters were changed to ki_NH3_min = 0.01, ki_NH3_max = 0.1, ki_NH4_min = 1.7, and ki_NH4_max = 3.1. For m3 the default parameters were used. The residual slurry fraction was set to 0.95 for this simulation.

References

1. Buhlmann CH, Mickan BS, Jenkins SN, Tait S, Kahandawala TKA, Bahri PA. Ammonia stress on a resilient mesophilic anaerobic inoculum: Methane production, microbial community, and putative metabolic pathways. Bioresour Technol [Internet]. 2019;275(December 2018):70–7. Available from: https://doi.org/10.1016/j.biortech.2018.12.012
